# Competing Reaction Pathways in Gas-Phase Oxidation of C_6_H_6_ by Protonated H_2_O_2_

**DOI:** 10.1021/acs.jpca.4c03722

**Published:** 2024-11-25

**Authors:** Sverre Løyland, Einar Uggerud

**Affiliations:** Hylleraas Centre for Quantum Molecular Sciences, Department of Chemistry, University of Oslo, P.O. Box 1033, Blindern 0315 Oslo, Norway

## Abstract

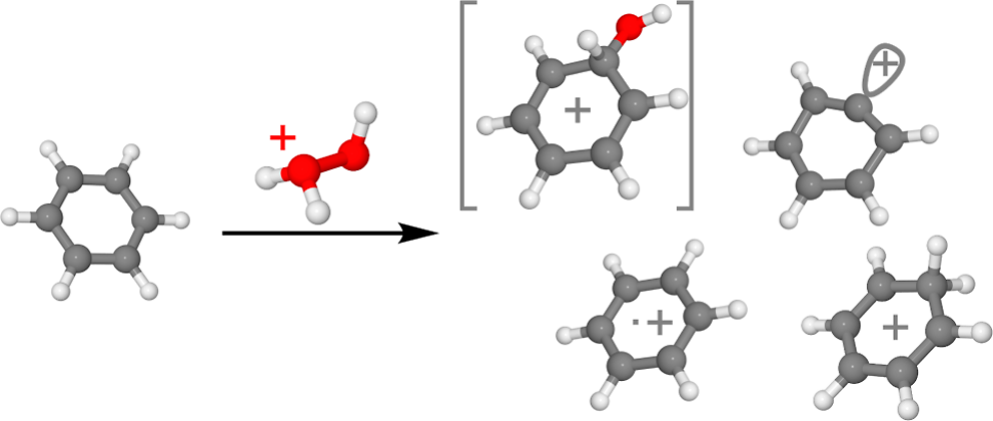

Reactions between protonated hydrogen peroxide and benzene (and benzene-*d*_6_) have been studied in the gas phase using an FT-ICR mass spectrometer. Four competing paths for the bimolecular system were identified, namely, proton transfer, hydride abstraction, dissociative single-electron transfer, and an electrophilic addition of HO^+^ to give the Wheland intermediate [C_6_H_6_, OH]^+^ followed by a subsequent elimination of water. The three latter pathways correspond to three different ways to oxidize benzene. All reaction mechanisms have been modeled using quantum chemical methods, and the calculations are in agreement with the experimental observations. The total reaction rate proceeds at collision rate (slightly higher than the calculated Langevin capture rate), which exemplifies the high reactivity of H_3_O_2_^+^ toward arenes. These observations demonstrate a much richer chemical landscape than previously inferred from the corresponding condensed phase reaction, where only electrophilic substitution by solvated HO^+^ was described.

## Introduction

Protonated hydrogen peroxide (**1**) is quite remarkable. It is an extremely potent oxidant and superelectrophile that acts as a source for singlet-state HO^+^ and can as such be regarded as monohydrated HO^+^ in its electronic singlet state as demonstrated by its gas-phase identity-reaction 1.^1^
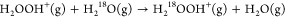
1H_3_O_2_^+^ reacts with a plethora of organic molecules, including unsubstituted alkanes and arenes to give alcohols.^2–4^ H_2_O_2_ is a much weaker base than H_2_O making superacidic conditions a necessity for the protonation of H_2_O_2_.^5^ The rather elusive H_3_O_2_^+^ ion has been characterized under these superacidic conditions by ^17^O NMR,^6^ and salts of H_3_O_2_^+^ have impressively been isolated from these solutions with weakly coordinating counterions and characterized by SC-XRD, IR, Raman, and NMR spectroscopy.^7,8^ In spite of its high reactivity, it can be reacted selectively, and its synthetic utility has been demonstrated.^9–17^ H_3_O_2_^+^ can be considered an ideal oxidizer because it can react selectively, gives water as the only byproduct, has a moderately good atom economy, does not require any metal catalyst and can accomplish late-stage C–H activations even though many substrates would be intolerant to the harsh conditions in a superacid.

Other sources of pseudo-HO^+^ are thought to play important roles in biological oxidation reactions, such as oxidation with ferryl heme compounds II with its reactive Fe^IV^OH moiety,^18,19^ and related synthetic reagents, such as Rozen’s HOF·MeCN oxygen-transfer reagent.^20–23^

H_3_O_2_^+^ ions have also been observed in the gas phase. The occurrence of H_3_O_2_^+^ in CH_4_/O_2_ microwave plasma suggests it plays a role in combustion processes^24^ and it has been suggested as a charge carrier in multiple charged water clusters, which may hold significance for the chemistry of the upper atmosphere.^25,26^ With H_2_O_2_ being observed in the interstellar medium,^27^ it is reasonable to assume it also reacts with abundant H_3_^+^ and forms (short-lived) H_3_O_2_^+^. This makes the H_3_O_2_^+^ ion of substantial theoretical interest and worthy of thorough mechanistic investigations.

Schreiner et al.^28^ compared the reactivity of protonated hydrogen peroxide and singlet state HO^+^ cations toward methane using computational methods. While they note that methane favorably undergoes electron transfer to ^1^HO^+^, they compare the stabilizing effect of the associated water on the electrophilic nature of ^1^HO^+^. They found that while ^1^HO^+^ inserts itself into the C–H bond without a barrier, H_3_O_2_^+^ needs to overcome a small barrier, but the identified product channels were the same. In both cases, the thermodynamic product was H_3_COH_2_^+^ and the kinetic product H_2_COH^+^ + H_2_. They further characterized the reactivity of the electrophiles based on their exothermicities as
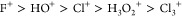


Several gas phase investigations of the reactivity of H_3_O_2_^+^ with aliphatic molecules have already been conducted in our lab, and it was determined that H_3_O_2_^+^ readily abstracts hydrides from alkanes to give carbocations,^29–31^ which are well known to hydrate to form alcohols according to reactions 2 and 3.
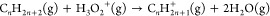
2
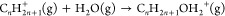
3

This is consistent with the observed solution phase reactivity, where alkanes are hydroxylated to form alcohols or derivatives of alcohols.

When H_3_O_2_^+^ is reacted with unsaturated alkenes in the condensed phase, epoxides are formed as in reaction 4(13) similar to reactions with peracids. The epoxides quickly undergo acid-catalyzed ring-opening under superacidic conditions (sln).
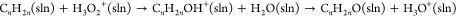
4

It is also known that H_3_O_2_^+^ readily hydroxylates arenes in solution to give phenols, presumably by electrophilic aromatic substitution, as shown in eq 5 according to the well-known addition/elimination scheme involving the so-called Wheland intermediate.^9–12,32,33^
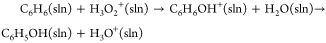
5

To our knowledge, no mechanistic investigation of this reaction has been conducted, and other mechanisms such as hydride abstraction or arene oxide intermediates analogous to the biochemical oxidation processes of arenes and reaction 4 appear likely.

To probe the extent of electrophilic aromatic substitution versus hydride abstraction, we devised an experiment in which C_6_D_6_ is reacted in the gas phase with H_3_O_2_^+^. If the major oxidation path is hydride (deuteride) abstraction, mostly C_6_D_5_^+^ is expected; however, if significant electrophilic aromatic substitution occurs, also C_6_D_4_H^+^ is expected to be formed as the result of scrambling of hydrogen and deuterium during the lifetime of a sufficiently long-lived Wheland intermediate.^34,35^

## Experimental Section

Protonated hydrogen peroxide was formed by CH_4_ chemical ionization of the complex of urea and H_2_O_2_ in an external ion source using a direct inlet probe. Commercially available [urea·H_2_O_2_] was contained in a small glass vial to limit contact with metal surfaces, which otherwise would have catalyzed its decomposition. The ions were injected into the cell of a 4.7 T Bruker APEX II FT-ICR mass spectrometer with either 2 × 10^–8^ mbar nominal partial pressure of C_6_H_6_ or C_6_D_6_ gas by leaking in the gas from a vial of the reactant through a leak valve. Benzene was dried with anhydrous MgSO_4_ and degassed by three freeze–pump–thaw cycles. All ions except H_3_O_2_^+^ were removed by correlated radio frequency sweeps and a pulse of helium was injected to cool the ions for 3.0 s. Another correlated radio frequency sweep was applied to remove any additional ions formed during the cooling, and the ions were allowed to react for 0 to 3 s before they were excited and detected. The background pressure in the cell was 2 × 10^–10^ mbar, which under CI conditions increased to 4 × 10^–9^ mbar. The total pressure in the cell in the experiments was thus about 2.4 × 10^–8^ mbar. During the helium pulse, the pressure increased to about 1 × 10^–6^ mbar before pumping back down to 2.4 × 10^–8^ mbar. 256 scans were averaged for each reaction delay. The FID was processed by zero-filling and applying an exponential apodization before applying the Fourier transform. The baseline noise was subtracted from the intensities, and the total intensity of identified ions normalized. The complete processing is shown in the Supporting Information.

The quantum chemical computations were performed by using the Gaussian 16 program suite. All calculations were performed by using DFT with the B3LYP functional and aug-cc-pVTZ basis set. All structures were optimized to either equilibrium positions or saddle points with one imaginary frequency. IRC calculations were performed from the transition states to confirm the equilibrium structures they connect. The zero-point vibrational energies were calculated by using the harmonic approximation. Some computed properties (proton affinities, hydride affinities, and ionization energies) of the relevant molecules and ions are summarized and compared to experimental values (except for hydride affinities where the experimental values have not been reported) in Tables S1 and S2 in the Supporting Information. Our computational investigation is focused on explaining the observed reactivity qualitatively and not necessarily as accurately as possible. The computed and experimental values in the Supporting Information show a reasonable agreement, but the computational results should be interpreted merely qualitatively.

## Results

**Figure 1:**
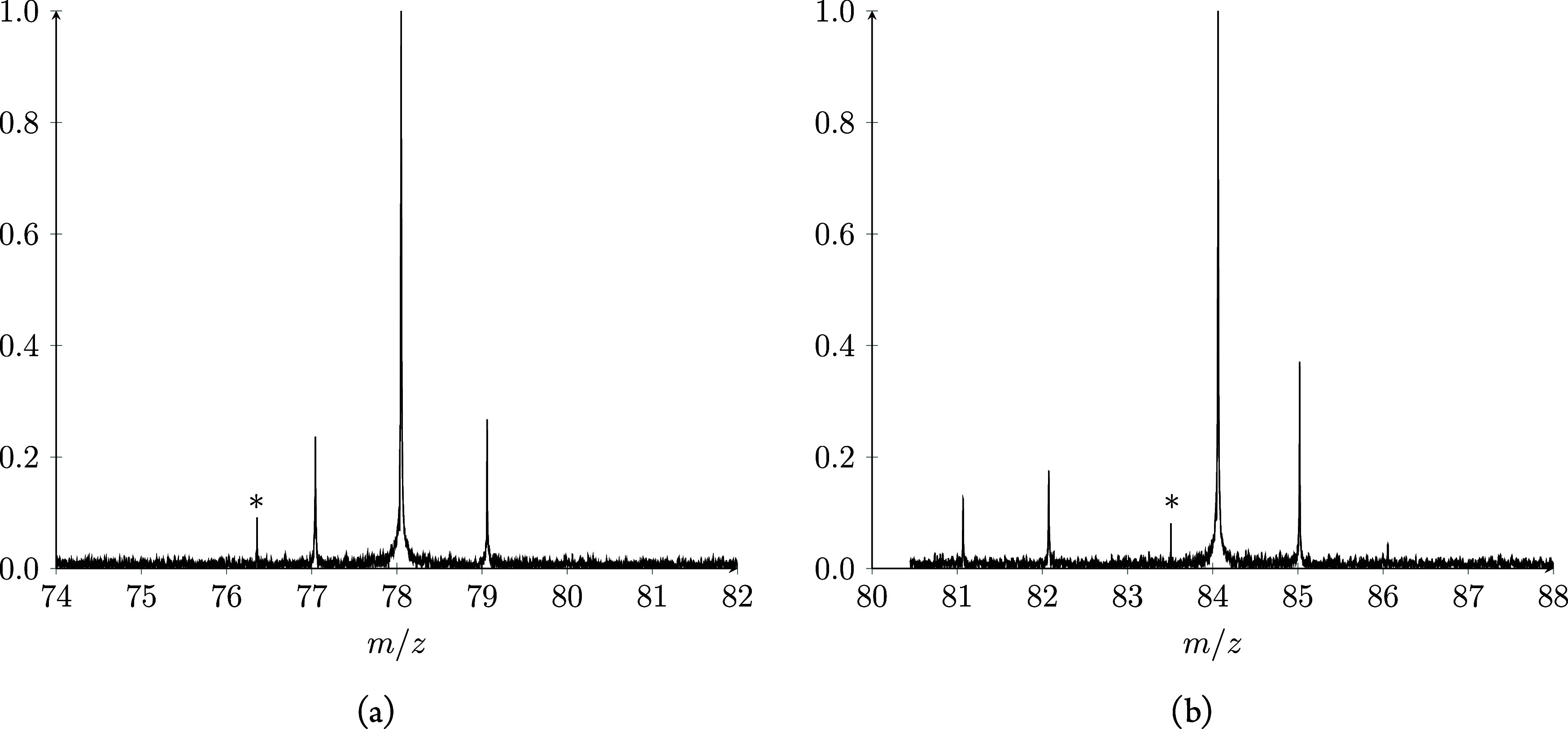
Product ion spectra obtained after the reaction between H_3_O_2_^+^ and benzene for 3 s. (a) Reaction with C_6_H_6_. The peak at *m*/*z* = 76.35 labeled with a star (*) is a 954 kHz electronic noise signal. (b) Reaction with C_6_D_6_. The peak at *m*/*z* = 83.52 labeled with a star (*) is a 872 kHz electronic noise signal.

When H_3_O_2_^+^ (**1**) is reacted with C_6_H_6_ (**2**), inside the FT-ICR cell, three product peaks at *m*/*z* = 77, 78, and 79 were detected, aside from the reactant peak at *m*/*z* = 35, corresponding to C_6_H_5_^+^ (**3**), C_6_H_6_^•+^ (**4**), and C_6_H_7_^+^ (**5**), respectively. The product ion spectrum is shown in Figure 1a. No other peaks were observed, neither products caused by unimolecular dissociation of H_3_O_2_^+^, where H_3_O^+^ + ^3^O is the major dissociation channel via a low-lying intersystem crossing to the triplet surface,^36,37^ reactions with trace background gases, nor adducts of the reactants. We propose the products are formed by proton transfer, dissociative single-electron transfer, hydride abstraction, and an electrophilic addition–elimination mechanism; as seen below.

### Proton Transfer

The major contribution to the peak at *m*/*z* = 79 is due to C_6_H_7_^+^ (**5**). The ion can be formed by a simple proton transfer as benzene has a significantly higher proton affinity (750.4 kJ mol^–1^) than hydrogen peroxide (674.5 kJ mol^–1^).^38^
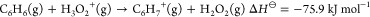
6

**Figure 2:**
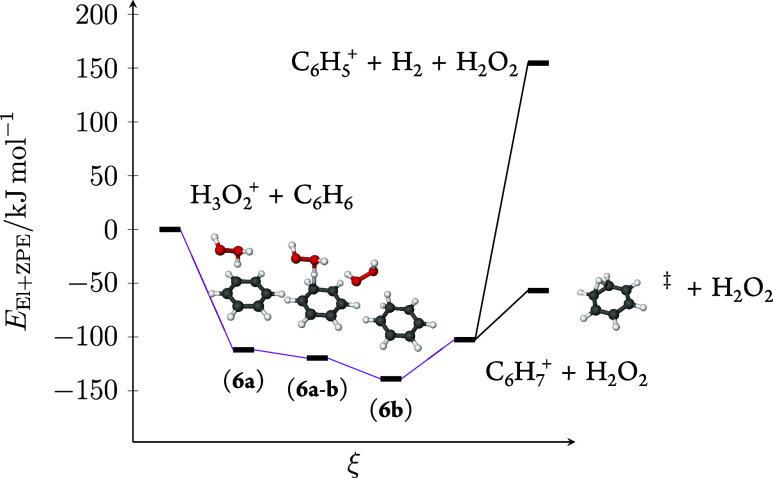
Computed potential energy surface with zero-point vibrational energy for the H_3_O_2_^+^ + C_6_H_6_ proton-transfer reaction and subsequent hydrogen elimination. The double dagger symbol (‡) indicates the transition-state geometry of a degenerate rearrangement.

Our quantum chemical computations show proton transfer proceeds without internal barriers via the weakly bonded ion-neutral intermediate complex H_2_O_2_···C_6_H_7_^+^ (**6b**). The computed transition state for proton transfer (**6a**–**b**) is lower in energy than **6a** because the difference in zero-point vibrational energy contribution between **6a**–**b** and **6a** is greater than the difference in purely electronic energy. After proton transfer has occurred, the adduct (**6b**) is readily dissociated to give free H_2_O_2_ and C_6_H_7_^+^ requiring 36 kJ mol^–1^. The calculated potential energy surface is illustrated in Figure 2. As this is the only reaction channel where benzene is not oxidized, we will devote the rest of the discussion to the other product peaks.

When the H_2_O_2_ product dissociates from C_6_H_7_^+^, the 103 kJ mol^–1^ excess energy will be distributed as kinetic and internal energy in the products. A crude model for estimating the energy distribution of the products is an equipartition of the energy among all of the 3*n* – 6 = 45 vibrational modes in the products. This leaves the C_6_H_7_^+^ ions with 75 kJ mol^–1^ of internal energy, which is sufficient to surpass the barrier (**5**–**5**) of 46 kJ mol^–1^ for essentially free proton mobility within C_6_H_7_^+^.

Although C_6_H_7_^+^ in principle could eliminate H_2_ to form C_6_H_5_^+^, our calculations show that this route is strongly endothermic. This was confirmed in separate control experiments in which proton transfer reactions between C_6_H_6_/C_6_D_6_ and CH_5_^+^ (PA = 544 kJ mol^–1^) and C_2_H_5_^+^ (PA = 681 kJ mol^–1^). In neither cases were any C_6_H_5_^+^/C_6_D_5_^+^ observed nor loss of D_2_ from C_6_D_6_H^+^ to give C_6_D_4_H^+^, which would be expected by the hydrogen scrambling described above even though the reaction with CH_5_^+^ is less endothermic than in the case of H_3_O_2_^+^.
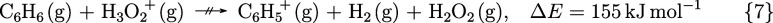


### Dissociative Single-Electron Transfer

The most intense signal at *m*/*z* = 78 is primarily due to the C_6_H_6_^•^+ (**4**) ion. We suggest it is formed by dissociative single-electron transfer from C_6_H_6_ to H_3_O_2_^+^, to give one water and one hydroxyl radical according to the process.
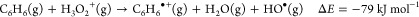
8

**Figure 3:**
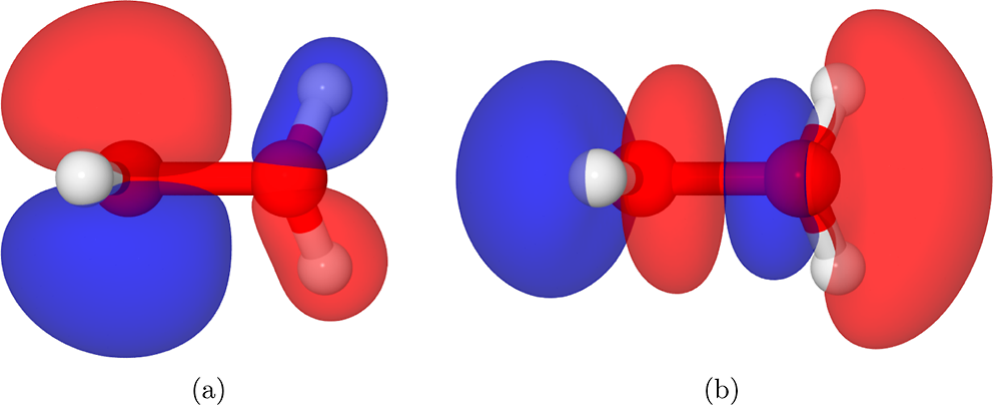
Canonical orbital 0.03 isosurfaces of protonated hydrogen peroxide. (a) HOMO. (b) LUMO.

The HOMO and LUMO of H_3_O_2_^+^ are illustrated in Figure 3. The LUMO strongly resembles a σ* orbital with a nodal plane approximately perpendicular to the O–O bond, suggesting the bond will be weakened or broken upon electron capture.

**Figure 4:**
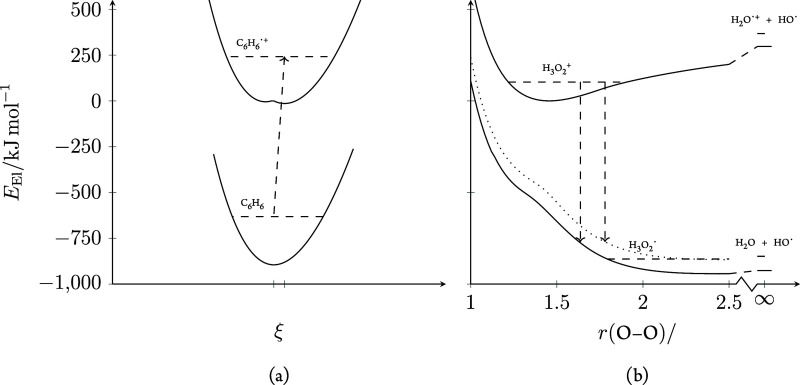
Depiction of the energetics involved in the dissociative electron transfer from C_6_H_6_ to H_3_O_2_^+^. The relative energy axis is the same for both plots. Solid lines depict electronic energy and dashed lines indicate zero-point vibrational energy. (a) Scan of electronic energy of C_6_H_6_ (lower) and C_6_H_6_^•+^ (upper) at interpolated and extrapolated structures. Dashed arrow shows adiabatic ionization. (b) Electronic energy of H_3_O_2_^+^ (upper), and H_3_O_2_^•^ (lower). The black dotted line shows the H_3_O_2_^•^ sum of electronic and projected zero-point vibrational energy at the frozen H_3_O_2_^+^ geometry. The arrow shows where the electron affinity is equal to the adiabatic benzene ionization energy.

A vertical single-electron transfer to H_3_O_2_^+^ in its equilibrium geometry involves insufficient energy to ionize benzene adiabatically. However, as the H_3_O_2_^•^ surface is practically dissociative, a slight elongation of the O–O bond greatly increases the electron accepting ability of H_3_O_2_^+^, as shown in Figure 4b to the extent where it surpasses the minimal adiabatic ionization energy of benzene illustrated in Figure 4a, while the energy necessary for the O–O perturbation remains lower than the zero-point vibrational energy. According to the computations, an elongation from an equilibrium of 1.46 Å to at least 1.78 Å is required.

The details of the single-electron-transfer process are without question very complex where the Born–Oppenheimer and adiabatic approximation breaks down and is more appropriately modeled by the Landau–Zener theory.^39–41^ Because it is difficult to estimate the electron-transfer rate quantitatively from first-principles, we limit ourselves to a qualitative consideration of the process.

As C_6_H_6_^•+^ only features a small Jahn–Teller distortion at an equilibrium geometry compared to C_6_H_6_, as shown in Figure 4a,^42,43^ the adiabatic ionization energy is close to vertical. The Franck–Condon factors for the transition from HOMO orbitals to diffuse Rydberg states that can overlap the H_3_O_2_^+^ acceptor at relatively large distances are presumably substantial and will thus give a fast reaction where the rate-limiting factor is the capture rate between H_3_O_2_^+^ and C_6_H_6_. This is consistent with C_6_H_6_^•+^ being the major product observed.

After electron capture, H_3_O_2_^•^ dissociates to H_2_O and HO^•^ as the excess energy surpasses the almost negligible binding energy of 14 kJ mol^–1^ of the transient H_2_O···HO^•^ adduct.

While benzene is oxidized in a single-electron transfer, no oxygen incorporation to form phenol is observed, most likely because the electron transfer happens at too long a distance preventing the formation of either a [C_6_H_6_, H_3_O_2_]^+^ or a [C_6_H_6_, HO]^+^ adduct where atoms can be exchanged and the energy liberated in the electron transfer is sufficient to dissociate the system to C_6_H_6_^•+^ + H_2_O + HO^•^.

### Hydride Abstraction and Electrophilic Addition–Elimination

**Figure 5:**
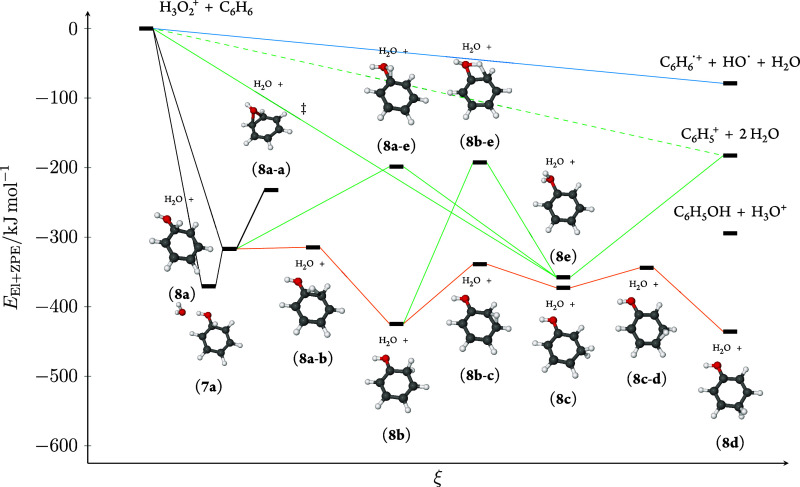
Computed potential energy surface with zero-point vibrational energy for the H_3_O_2_^+^ + C_6_H_6_ system. The dissociative electron transfer is colored blue, the hydride abstraction green, and the addition path is orange. The double dagger symbol (‡) indicates the transition state of a degenerate rearrangement.

The *m*/*z* = 77 signal is due to the C_6_H_5_^+^ (**3**) product ion, which may be produced in two distinct ways. The complexity of the mechanism is evident in the reaction between H_3_O_2_^+^ and C_6_D_6_, which yields both C_6_D_5_^+^ (**3**-*d*_5_) and C_6_D_4_H^+^ (**3**-*d*_4_), as shown in Figure 1b, in addition to C_6_D_6_^•+^ and C_6_D_6_H^+^. The calculated potential energy surface is shown in Figure 5.

The simplest path to C_6_H_5_^+^ (**3**) is a direct hydride abstraction by H_3_O_2_^+^ to give the phenyl cation plus two water molecules. Our calculations indicate that this is a direct mechanism that proceeds without passing through an intermediate energy minimum. Furthermore, as no H_3_O^+^ is observed, even though this is the thermodynamically most favorable dissociation channel, this shows that any coordinated water, as in **7**, dissociates quickly before any proton transfer to the leaving water molecule can occur. This is also consistent with the observations for reactions between alkanes and H_3_O_2_^+^ in which no H_3_O^+^ was observed either.^29^ Applying the simple equipartition model for the distribution of internal energy for the [C_6_H_6_, H_3_O_2_]^+^ system gives that after dissociating the initial H_2_O with a binding energy of about 85 kJ mol^–1^ in the case of **7a** → **8a**, the 333 kJ mol^–1^ internal energy is partitioned such that 267 kJ mol^–1^ internal energy is left in the C_6_H_6_OH^+^ ion, still more than enough to dissociate the second H_2_O.
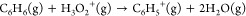
9

The second path to hydride abstraction is an indirect addition/elimination mechanism. Following this mechanism the HO^+^ moiety of H_3_O_2_^+^ first adds to the ring upon loss of the first H_2_O molecule giving rise to the covalently bonded (Wheland type) C_6_H_6_OH^+^ (**8a**) intermediate. During the lifetime of this intermediate the ipso hydrogen may migrate to the ortho position to give **8b**, and also further to the meta and para positions, to give **8c** and **8d**, respectively. This ring walk mechanism allows, in principle, for exchange of the positional identity of the ring hydrogens. In order for also the hydroxyl H to equilibrate with the ring hydrogens, it is necessary to also involve a fifth isomer, **8e** by multiple passages of the transition states associated with the geometries **8a**–**e** and/or **8b**–**e**. Please note that both transition states lie close in energy to the dissociated final products C_6_H_5_^+^ + 2H_2_O. Loss of the second H_2_O from the manifold of the Wheland intermediates also occurs from **8e**.

In reactions with benzene-*d*_6_, there are two possibilities
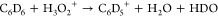
10
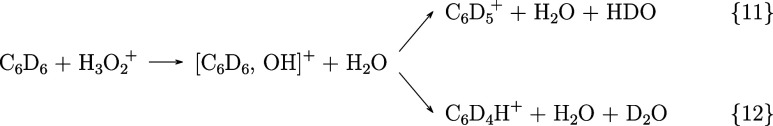
11

It is difficult for us to assess to which extent each of the three pathways contribute and how efficiently [C_6_D_6_, OH]^+^ scramble the H and D. If scrambling in the intermediates along the indirect path is complete and any isotope effects are ignored, the expected relative neutral loss of D_2_O to HDO is
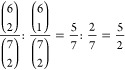
However, the observed ratio is 0.6(2), which shows that either the direct path is more important for the gas-phase reaction or the hydrogens/deuteriums are not completely scrambled in the lifetime of the intermediate during the indirect mechanism.

**Figure 6:**
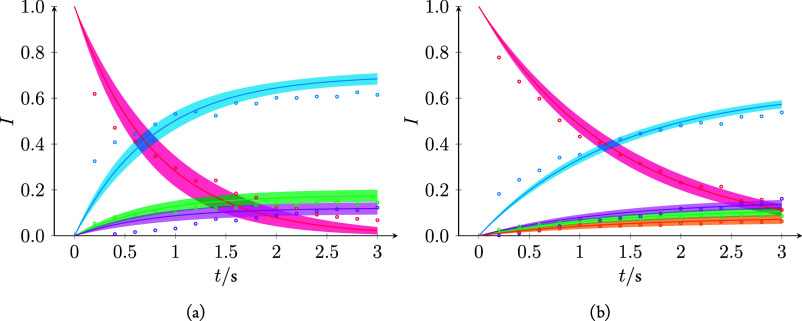
Relative intensities (circles) and model fit (lines) with one standard error confidence band (68% confidence) not accounting for pressure uncertainty. (a) Isotope corrected abundances for H_3_O_2_^+^, C_6_H_5_^+^, C_6_H_6_^+^, and C_6_H_7_^+^ in red, green, blue, and purple, respectively. (b) Isotope corrected abundances for H_3_O_2_^+^, C_6_D_4_H^+^, C_6_D_5_^+^, C_6_D_6_^+^, and C_6_D_6_H^+^ in red, orange, green, blue, and purple, respectively.

The reactions between H_3_O_2_^+^ and C_6_H_6_/C_6_D_6_ were performed with varying reaction times to determine the kinetics more accurately and a simple competitive first-order kinetic model was devised. The rate coefficients of the model were fitted to the data to give the rates, as shown in Table 1. The data and model fit are listed in Figure 6.

**Table 1:** Rate Coefficients, *k*, and Branching Ratios, *f*, for the Reactions of H_3_O_2_^+^ with C_6_H_6_ and C_6_D_6_ with the Langevin Capture Rate *k*_L_

H_3_O_2_^+^ + C_6_H_6_			H_3_O_2_^+^ + C_6_D^6^		
product	*k*/(10^–10^ cm^3^ s^–1^)	*f*	product	*k*/(10^–10^ cm^3^ s^–1^)	*f*
C_6_H_7_^+^	6(4)	0.12(3)	C_6_D_6_H^+^	4(3)	0.15(3)
C_6_H_6_^•+^	34(21)	0.70(6)	C_6_D_6_^+^	18(11)	0.65(3)
C_6_H_5_^+^	9(6)	0.18(4)	C_6_D_5_^+^	3(2)	0.12(2)
			C_6_D_4_H^+^	2(2)	0.08(2)
*k*_tot_	49(30)		*k*_tot_	28(17)	
*k*_L_	15		*k*_L_	15	

The greatest uncertainty in the measurement of the rate coefficient comes from the pressure measurement of benzene. The nominal pressure was measured using a cold-cathode ionization gauge reading 2.0 × 10^–8^ mbar in all experiments which, upon applying a benzene correction factor of 5.90, gives a pressure of 3.4 × 10^–9^ mbar. Additionally, the pressure was calibrated by performing the proton-transfer reactions
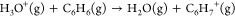
13
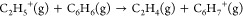
14with known rate coefficients of 1.9 × 10^–9^ cm^3^/s^44–48^ and 3.0 × 10^–9^ cm^3^/s^49^ from the literature gave pressures of 1.1 × 10^–8^ and 4.3 × 10^–9^ mbar, respectively. While the ionization gauge is sensitive to the conditions and only accurate to 5% under ideal conditions, the pressure discrepancies in the calibration reactions indicate an inconsistency in the rate coefficients reported in the literature or in our experiment. While the benzene pressure in the cell could not be determined accurately, it is clear that the observed rates are of the order of the Langevin capture rate.

Although the total reaction rate for C_6_H_6_ appear about twice that for C_6_D_6_, the difference is not statistically significant when the pressure uncertainty is taken into account. Separate control experiments in which C_6_H_6_ and C_6_D_6_ were introduced together in the reaction cell and the ratios adjusted to give approximately equal intensities of their molecular ions with internal 70 eV EI also gave approximately the same intensities when H_3_O_2_^+^ was subsequently introduced into the cell. The plots are shown in the Supporting Information. The difference in appearance energies of the molecular ions of C_6_H_6_ and C_6_D_6_ are about 30^50^ and 45 meV difference in ionization energy as previously shown by photoionization spectroscopy.

The fact that the measured H_3_O_2_^+^ intensity falls slower than the model predicts at longer reaction times in Figure 6a together with an overestimation of the C_6_H_6_^•+^ rate and that C_6_H_7_^+^ appears to have a late onset may suggest that the ions are not completely thermalized leading to a biexponential rate law. However, the ions could not be further thermalized without losing too much signal. Attempts at using argon for cooling the ions were performed analogous to earlier experiments in our lab.^29,37^ As argon is heavier and more polarizable than helium, it usually cools the ions more efficiently, but as each collision is more energetic compared to helium, too much fragmentation was observed. This was not a problem in earlier experiments, where the neutrals were less reactive toward the protonated hydrogen peroxide and not as much intensity of H_3_O_2_^+^ was lost during the cooling process. The late onset can potentially also be explained by C_6_H_7_^+^ being a secondary product formed by, for example, hydrogen atom transfer to C_6_H_6_^•+^ from a background gas. The reactions are endothermic, which eliminates C_6_H_6_ as a possible partner in a third collision. Fitting a model in which C_6_H_6_^•+^ reacts with a background gas in a first-order reaction, shown in the Supporting Information, gives a rate *kp*_bg_ = 3 × 10^–10^ cm^3^s^–1^ mbar, much higher than what can be reasonably expected based on the background pressure and Langevin capture rate.
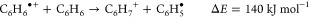
15
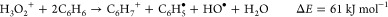
16

Another possible explanation for the deviation from the model is that the initial distribution of ions at *t* = 0 s is not only H_3_O_2_^+^ but also minor amounts of the product ions formed during the thermalization event, which the final cleanup sweep did not eject from the cell. Adding parameters for nonzero initial intensities significantly improves the fit at the cost of having additional parameters to account for. While the rate coefficients for the proton transfer and hydride transfer do not change significantly with the different models tested, the rate coefficient for electron transfer drops from 3.4(6)×10^–9^ to 1.4 to 2.4 cm^3^ s^–1^ depending on the model as shown in the Supporting Information. In control spectra acquired at *t* = 0 s, only H_3_O_2_^+^ was detected which undermines the models with nonzero initial product intensities.

Measurements of the total reaction rate of 5(3)×10^–9^ cm^3^ s^–1^ for C_6_H_6_ and 3(2)×10^–9^ cm^3^ s^–1^ for C_6_D_6_ reveals a rate coefficient in excess of the Langevin capture rate of 1.5 × 10^–9^ cm^3^ s^–1^ (for both C_6_H_6_ and C_6_D_6_) indicating a very fast reaction.^51^

While the absolute rate coefficient could not be determined sufficiently accurately, the branching ratio of each reaction route can be determined more accurately, as they do not depend on the pressure. The rate coefficients and branching ratios are tabulated in Table 1. Assuming that the mechanisms for hydride/deuteride loss are only hydride (deuteride) transfer with no scrambling and an addition—elimination mechanism with complete scrambling, the rate coefficients for each of the mechanisms can be derived and are shown as hydrogen abstraction and addition—elimination in Table 1.

The experimental conditions did not allow the [^13^CC_5_H_6_^•+^] = 79.050 and [C_6_H_7_^+^] = 79.054 peaks to be completely resolved which also means a determination based on peak height will miss some abundance. With peak widths of about 100 Hz corresponding to a width of Δ*m*/*z* = 0.005 in the *m*/*z* = 79 range, most of the abundance of the less abundant ion will be lost when using peak heights. This may explain the observed deviation in peak height for *m*/*z* = 79 from the model. When integrals were attempted to alleviate this problem, the integrals turned out to be affected too much by the baseline noise.

We also acknowledge the similarity in the products formed in the reactions studied to the products formed in the chemical ionization of C_6_H_6_ and C_6_D_6_ with methane as the reagent gas. This raises the concern of whether highly reactive ions formed from chemical ionization of methane can contribute to the observed reactivity either by a small amount avoiding ejection from the cell or by a small amount leaking into the cell after ejection. Control experiments in which no hydrogen peroxide—urea adduct was introduced into the ion source gave no product signals, and experiments in which CH_5_^+^ or C_2_H_5_^+^ were isolated and reacted with benzene gave the same products as in the reaction with H_3_O_2_^+^, but also gave fragmentation products of benzene, such as C_4_H_4_^•+^, which was not observed in the reaction with H_3_O_2_^+^ and the product distributions were different.

## Discussion

While our experiments have shown new dissociative single-electron transfer reactivity for H_3_O_2_^+^ and a new hydride abstraction path in the case of reactions with benzene in the gas phase, it still remains an open question whether this reaction also occurs in solutions. Analogous solution phase experiments to our hydride shift experiments for C_6_D_6_ will likely be unfruitful as benzene is readily protonated and exchanges protons/deuterons under the strongly acidic conditions necessary for the formation of H_3_O_2_^+^. The presence of transient HO^•^ and C_6_H_6_^•+^ in solution can, however, be monitored using EPR spectroscopy. Similar single-electron transfers may have been overlooked in other reactions with HO^+^ sources.

If a single-electron transfer occurs in the condensed phase where the dissociating system is confined in the solvent structure and the energy can be dissipated to the surroundings quickly, the benzene radical cation and hydroxyl radical could successively recombine to form protonated phenol by electrophilic addition of HO^•^ to the benzene cation. Alternatively, HO^•^ can abstract a hydrogen atom to form the C_6_H_5_^+^ phenylium ion, which then can be hydrated. The hydration of the phenylium cation to form protonated phenol tautomers has been studied.^52,53^ While the C_6_H_6_^•+^ ion is not reactive toward single water molecules or the smallest water clusters in the gas phase, it has been found that it is reactive toward water clusters (H_2_O)^*n*^ of size *n* ≥ 4, where it will transfer a proton to the water cluster to form C_6_H_5_^•^.^54^ It has also been shown in matrix isolation studies that C_6_H_5_^•^ can react with water to give a hydroxyl radical that can attack the benzene ring to form phenol.^55,56^ To our knowledge, a single-electron transfer mechanism for the formation of phenols by H_3_O_2_^+^ has not been considered to this end. It appears that such gas-phase reactions, in principle, are also possible in the condensed phase.

Our kinetic experiments show rate coefficients that are in slight excess of the Langevin capture rate. Although the Langevin rate is well within the estimated uncertainty, there is good reason to assume that the rate exceeds the Langevin limit. The simple Langevin capture model only takes charge-induced dipole interaction into consideration and is often extended with the permanent dipole of the neutral in models, such as the average and locked dipole orientation models to give slightly higher capture rates. Benzene does not feature a permanent dipole but a large quadrupole moment. Analogous extensions of the Langevin model for permanent quadrupoles belonging to the *D*_∞*h*_ group has been derived, and the average quadrupole orientation theory has shown excellent agreement with experiments for the proton (deuteron) transfer reaction 17 with rate coefficients in slight excess of the Langevin capture rate.
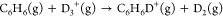
17The H_3_O_2_^+^ ion also has an appreciable dipole moment, which also contributes somewhat to increase the capture rate.

We also acknowledge the possibility that the reaction cross-section for electron transfer exceeds the Langevin rate. The electron-transfer process does not require the same proximity between the reactants that is necessary for the formation of an adduct, and it is adequate that the orbitals between the reactants overlap sufficiently for a short period of time. While our experiments show significant uncertainty in the measured rate coefficients caused mainly by the uncertainty in the benzene pressure, it is reasonable to expect the true rate coefficient to be larger than the Langevin rate.

Furthermore, Gunawardena et al.^39^ discuss the electron transfer rate versus the proton transfer rate within the Landau–Zener transition model and show that electron transfer reactions can be kinetically favored even when proton transfer is thermodynamically favored which also appears to be the case in the reaction between H_3_O_2_^+^ and benzene. This is because electron transfer can occur while the reactants are at a significantly greater distance than reactions requiring the transfer of atoms without the necessity for the formation of a prereactive complex.

It has also been suggested that energy resonance effects may be of importance for charge transfer processes in the gas-phase where a lower difference in ionization and recombination energies give rise to increased electron transfer rates.^57–59^ Because the electron affinity of H_3_O_2_^+^ varies wildly with the O–O distance and benzene having a reasonable density of states being a polyatomic molecule, this resonance criterion should easily be met.

It is interesting to compare the monohydrated HO^+^ in the form of H_3_O_2_^+^ against the naked HO^+^ ion. While H_3_O_2_^+^ is a ground-state singlet, HO^+^ has a triplet ground-state. Even though HO^+^ is easily formed by electron ionization of H_2_O in mass spectrometers and is a molecule of astrochemical relevance, not many reactions involving the ion have been studied. Attempts were made at reacting HO^+^ with benzene, but all of the HO^+^ signal intensity was lost during the thermalization event. When the highly nonthermal HO^+^ was reacted with benzene, a product ion spectrum similar to the EI spectrum of benzene was observed in addition to the reactant HO^+^ signal and a H_2_O^•+^ product signal. The high-energy HO^+^ reaction is not instructive to compare with the thermal H_3_O_2_^+^ reaction. The thermalization of HO^+^ ions should be possible in a separate ion trap where the ions can subsequently be injected into a trap containing benzene vapor, but our instrumentation does not have this capability. We believe the thermal reaction between HO^+^ in either its triplet ground state or excited triplet state will initially yield almost only C_6_H_6_^•+^ in a reaction with benzene as the electron affinity at equilibrium geometry of both the singlet and triplet state HO^+^ ions far exceed the ionization energy of benzene. Because the HO^+^ ions do not require a geometrical perturbation like H_3_O_2_^+^ to undergo electron transfer, it is more likely that it will happen at a great distance without the formation of an ion-neutral complex where atoms may be exchanged. Because the density of states generally grows with increasing energy, it is also likely that near-resonant energy transfer can occur. However, the electron affinity of singlet and triplet HO^+^ is larger than the appearance energy of several fragment ions of benzene,^60,61^ which may give rise to secondary ions without formation of any H_2_O^•+^.

It appears there are only two cases where HO^+^ and H_3_O_2_^+^ have been reacted (experimentally) with the same compound, namely the alkanes methane^37,62^ and ethane.^37,63^ When HO^+^ reacts with CD_4_, the two products CD_4_^•+^ (88%) and HD_2_O^+^ (12%) are formed^62^ which is in stark contrast to H_3_O_2_^+^, which is unreactive toward methane.^37^ While a single-electron transfer to H_3_O_2_^+^ is endothermic, hydride abstraction is exothermic and the existence of a barrier will hinder the reaction under thermal conditions. In the reaction between naked HO^+^ and C_2_H_6_, the five products H_3_O^+^ (10%), C_2_H_4_^+^ (65%), C_2_H_5_^+^ (20%), C_2_H_6_^+^ (3%), and C_2_H_7_^+^ (2%) are observed,^63^ while in the reaction H_3_O_2_^+^ + C_2_D_6_, the products C_2_H_5_^+^ (97%) and HD_2_O^+^ (3%).^37^ It appears HO^+^ favors electron transfers more strongly than H_3_O_2_^+^ presumably as it has a stronger energetic driving force and does not need to undergo significant geometric perturbation to allow for an electron transfer. HO^+^ also has a much stronger hydride affinity than H_3_O_2_^+^, but the triplet ground-state of HO^+^ necessitates a spin-inversion for the hydride abstraction which may be slow while H_3_O_2_^+^ yielding two equivalents of water has a strong entropic driving force. The proton affinity of H_2_O_2_ is also much greater than O which makes HO^+^ more acidic than H_3_O_2_^+^, although this is not evident from the reactions with methane and ethane. This shows that although H_3_O_2_^+^ can undergo H_2_O-exchange as in reaction 1, it shows a marked difference in reactivity toward the simplest alkanes compared to HO^+^.

## Conclusions

The gas-phase reactivity of H_3_O_2_^+^ toward C_6_H_6_ and C_6_D_6_ has been explored, which revealed three oxidation pathways in addition to a proton-transfer channel. While electrophilic addition of HO^+^ has previously been described for the solution phase system and hydride abstraction in the gas-phase reaction with alkanes, a new dissociative single-electron transfer has been discovered, which may hold implications for the reactivity of HO^+^ sources in both condensed and gaseous states. The calculated submerged barriers are in agreement with the fast kinetics observed, and the high rate coefficients for the oxidation paths underpin the powerful oxidative properties of H_3_O_2_^+^ and demonstrate its potential use in oxidation of arenes for both synthetic uses as well use in degradation processes.

## Data Availability

http:doi.org/10.6084/m9.figshare.25879024: Downloadable FT-ICR-MS data sets and data processing script. http:doi.org/10.19061/iochem-bd-6-366: Online database of quantum chemical computations.

## Supporting Information Available

Document with additional mass spectra, data processing script, and data for quantum chemical computations (PDF)
