# Supplementary material for: Competing Reaction Pathways in Gas-Phase Oxidation of C6H6 by Protonated H2O2
Source: J Phys Chem A. 2024 Nov 25;128(49):10465–73. doi: 10.1021/acs.jpca.4c03722 (PMC11647889; doi:10.1021/acs.jpca.4c03722)
Supplement: Supplementary file 1 — jp4c03722_si_001.pdf [file jp4c03722_si_001.pdf]

Supporting Information: Competing Reaction Pathways in Gas-Phase Oxidation of C_6H_6 by Protonated H_2O_2

Sverre Løyland and Einar Uggerud*

Hylleraas Centre for Quantum Molecular Sciences, Department of Chemistry, University of Oslo, P.O. Box 1033 Blindern, 0315 Oslo, Norway

E-mail: einar.uggerud@kjemi.uio.no

Phone: +47 22855537

Contents

S1 Gas-phase ion energetics summary **S2**

S2 Control experiments **S2**

S3 Potential energy surface **S3**

S4 Processing script **S4**

S5 Calculations **S32**

References **S39**

S1 Gas-phase ion energetics summary

Table S1: Experimental and computed proton affinities and ionization energies for water, hydrogen peroxide and benzene. References in superscript.

	H_2O	H_2O_2	C_6H_6
experimental PA/kJ mol^{-1}	691.0[S1]	674.5[S1]	750.4[S1]
computed PA/kJ mol^{-1}	674.5	653.3	754.3
experimental IE/eV	12.62[S2]	10.58[S3,S4]	9.24[S5]
computed IE/eV	12.60	10.55	9.04

Table S2: Computed hydride affinities for the singlet and triplet state hydroxylium cation and protonated hydrogen peroxide.

	$^1HO^+$	$^3HO^+$	$H_3O_2^+$
HA/kJ mol^{-1}	1955	1666	1343

S2 Control experiments

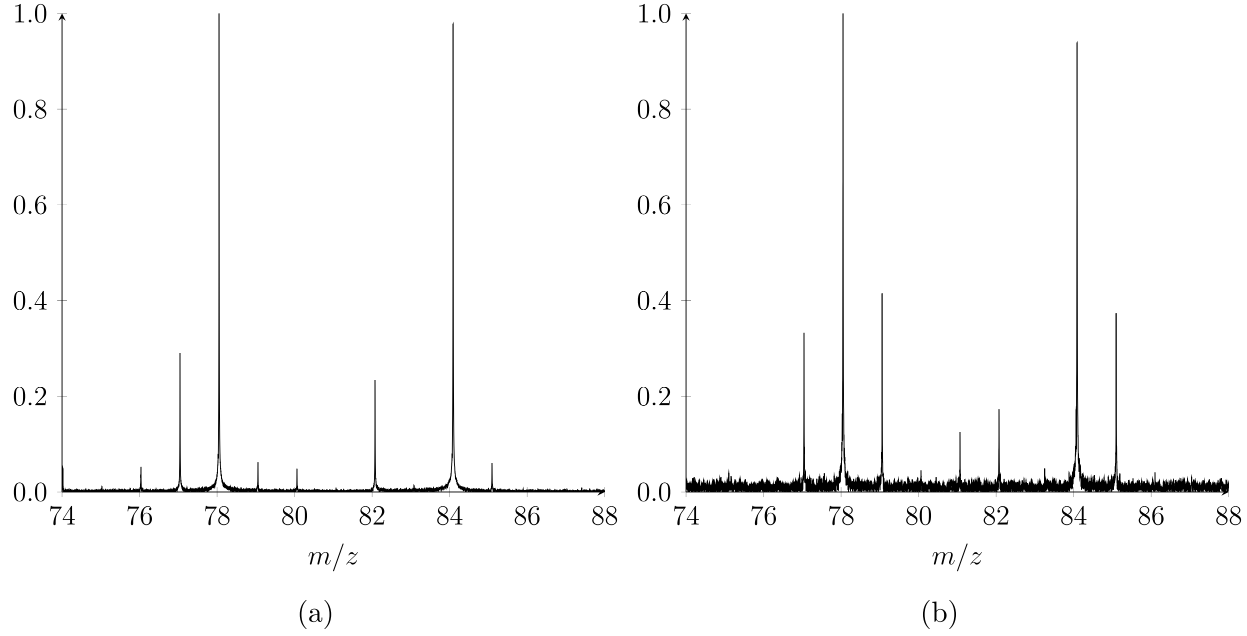

Figure S1: Control experiments with approximately equimolar C_6H_6/C_6D_6 mixtures at about 1×10^{-8} mbar nominal partial pressure each. (a) Internal EI spectrum. (b) Product spectrum after 3000 ms for the reaction with $H_3O_2^+$.

S3 Potential energy surface

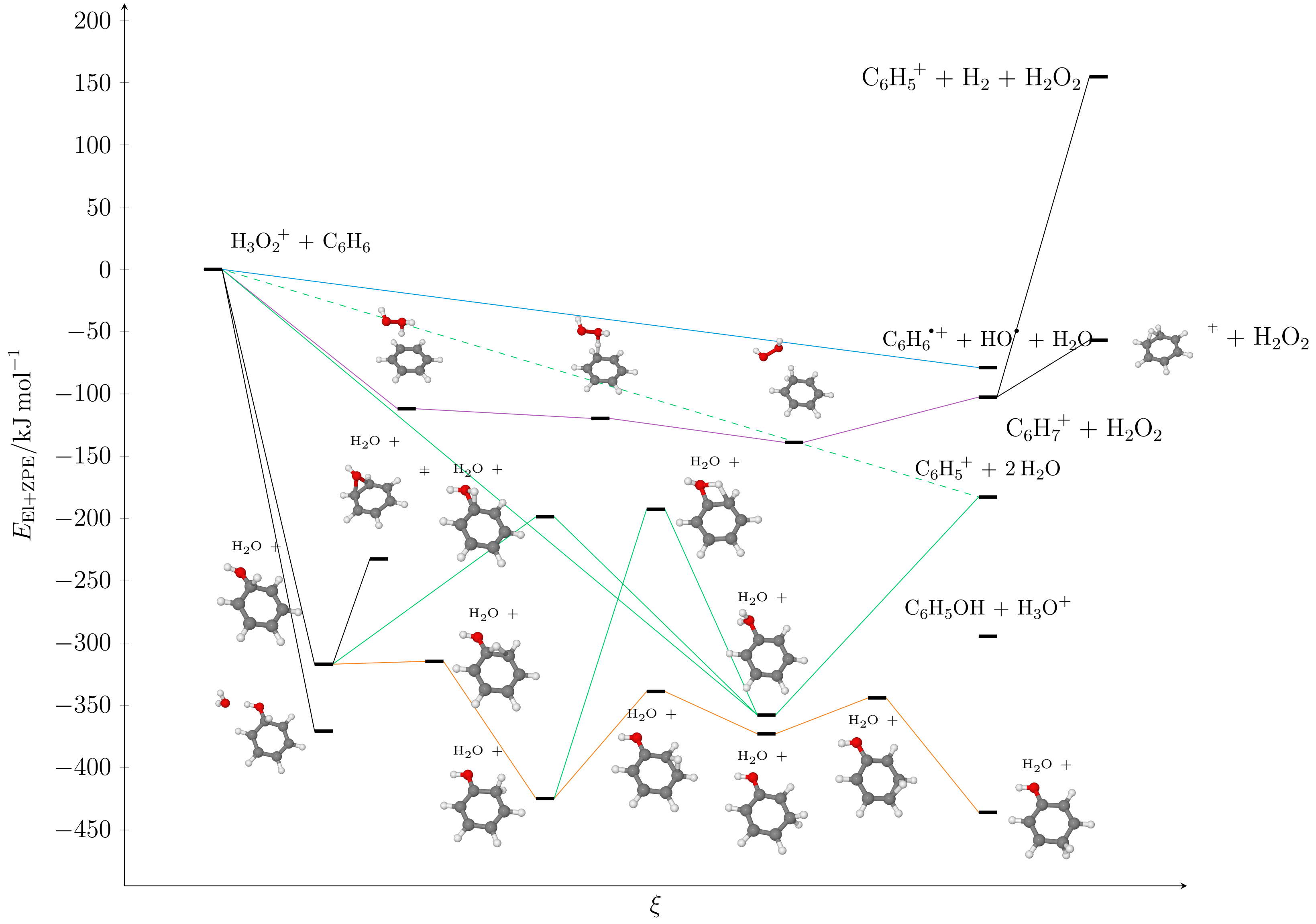

Figure S2: Computed potential energy surface with zero-point vibrational energy for the $H_3O_2^+$ + C_6H_6 proton transfer reaction and subsequent hydrogen elimination. The prton transfer is colored purple, the dissociative electron transfer blue, the hydride abstraction green and the addition path is orange. The double dagger symbol (‡) indicates the transition state of a degenerate rearrangement.

S4 Processing script

This notebook processes the raw FT-ICR data and extracts rate constants from kinetic plots.

Imports

Import all the modules to be used and display their versions

[1]:
```
import sys
import os
import re
import glob
import IPython.utils
print('Python version %s' % sys.version)

import numpy as np
print('Numpy version %s' % np.__version__)

import scipy as sp
import scipy.constants as cts
print('Scipy version %s' % sp.__version__)

import matplotlib as mpl
import matplotlib.pyplot as plt
from matplotlib.markers import MarkerStyle as ms
print('Matplotlib version %s' % mpl.__version__)

import symfit as sf
print('Symfit version %s' % sf.__version__)

import uncertainties as unc
print('Uncertainties version %s' % unc.__version__)

from isotope import isotopes as iso
print('Isotope version %s' % iso.version)

with IPython.utils.io.capture_output():
    import spike
    import spike.File.Apex0
    from spike.plugins import Peaks
    from spike.NPKData import parsezoom
print('Spike version %s' % spike.__version__)
```

```
Python version 3.11.9 | packaged by Anaconda, Inc. | (main, Apr 19 2024,
16:40:41) [MSC v.1916 64 bit (AMD64)]
Numpy version 1.26.4
Scipy version 1.12.0
Matplotlib version 3.8.4
Symfit version 0.5.6
Uncertainties version 3.1.7
Isotope version 1.1
Spike version 0.99.33
```

Plot settings

Make plots A4 size.

[2]:
```
plt.rc('figure', figsize=(11.69,8.27))
```

Make plots interactive.

[3]:
```
%matplotlib widget
```

Helper functions

These functions simplify the processing of the data sets.

Find FT-ICR experiments in root directory.

[4]:
```
def get_experiments(root, name='fid'):
    experiments = []
    for directory, _, _ in os.walk(root):
        path = glob.glob(os.path.join(directory, name))
        if path != [] and 'special' not in directory:
            experiments.append(directory)
    return experiments
```

Convert formulas to monoisotopic masses.

[5]:
```
iso._lexer = re.compile(r"[A-Z][a-z]*|\d+|\+|-|[()]|<EOS>") # Fix bug in isotope 1.1␣
↪module which would ignore charges, originally "[A-Z][a-z]*|\d+|\+|-|[()]|<EOS>"

iso.name_t["D"] = 0
m_e = cts.physical_constants['electron mass in u'][0]
m_d = cts.physical_constants['deuteron mass in u'][0]
iso.isotope_t[0].append(iso.Isotope(0, 2, m_d + m_e, 1.0)) # Add D as an element

def formulas_to_mz(formulas):
    mz = []
    for formula in formulas:
        mz.append(iso.parse_formula(formula).monoisotop())
    return mz
```

Process a FID by zero-filling, apodizing, applying FFT and using magnitude spectrum.

[6]:
```
def process_fid(data):
    data.zf(4).apod_em(40).rfft().modulus().set_unit('m/z')
    noise = np.median(data.buffer)
    data.set_buffer(data.buffer - noise)
    return noise
```

Get the abundance of a formula in an experiment by processing the FID and taking either the intensity or the integral of the corresponding peak.

[7]:
```
def get_abundances(formulas, experiments, mz_list, method='intensity',␣
↪detection_limit=3, mz_tol=0.05):
```

```
    abundances = np.full(len(experiments), np.nan, dtype=list(zip(['t',] + formulas,␣
↪'d'*(len(formulas)+1))))
    sigmas = np.full(len(experiments), np.inf, dtype=list(zip(['t',] + formulas,␣
↪'d'*(len(formulas)+1))))

    for i, experiment in enumerate(experiments):
        data = spike.File.Apex0.Import_1D(experiment + '/fid')
        noise = process_fid(data)

        abundances['t'][i] = get_reaction_delay(experiment + '/acqus')
        sigmas['t'][i] = abundances['t'][i]

        for formula, mz in zip(formulas, mz_list):
            z1, z2 = parsezoom(data, (mz - mz_tol, mz + mz_tol))
            buff = data.get_buffer()[z1:z2]
            idx = np.argmax(buff)
            idxtot = int(z1) + idx

            # Skip peak if at edge of range
            if idx == 0 or idx == len(buff) - 1:
                continue



            if method == 'intensity':
                abundance = buff[idx].real
            elif method == 'integral':
                peak = Peaks.Peak1D(0, data.axis1.itomz(idxtot), buff[idx].real, data.
↪axis1.itoix(idxtot))
                data.peaks = Peaks.Peak1DList(source=data)
                data.peaks.append(peak)
                data.centroid()
                data.integrate()
                abundance = data.integrals[0].value

            if abundance >= detection_limit * noise:
                abundances[formula][i] = abundance
                sigmas[formula][i] = 1

    return abundances, sigmas
```

Get the reaction delay from the acquisition parameters.

[8]:
```
def get_reaction_delay(acqus, idx=10):
    params = spike.File.Apex0.read_param(acqus)
    delays = params['$D']
    return float(delays[idx])
```

Normalize the peak abundances.

[9]:
```
def normalized(abundances):
    from numpy.lib.recfunctions import structured_to_unstructured as s_to_u
    from numpy.lib.recfunctions import unstructured_to_structured as u_to_s
```

```
    normalized_abundances = abundances.copy()
    normalized_abundances = u_to_s(np.
↪vstack((normalized_abundances['t'],(s_to_u(normalized_abundances)[:,1:].T / np.
↪sum(np.nan_to_num(s_to_u(normalized_abundances))[:,1:],axis=1)))).T,␣
↪dtype=normalized_abundances.dtype)
    return normalized_abundances
```

Format chemical formulas to use subscripts and superscripts.

```
[10]: def format_formula(formula):
          subscripted = re.sub(r'(\D*)(\d+)(\D*)', r'\1_{\2}\3', formula)
          superscripted = re.sub(r'(.*)([+-])', r'$\1^\2$', subscripted)
          return superscripted
```

Shotcut to find experiments, calculate monoisotopic masses, find peaks and normalize their intensities.

```
[11]: def get_normalized_abundances(directory, formulas, method='intensity',␣
↪detection_limit=3, mz_tol=0.05):
          experiments = get_experiments(directory)
          mz = formulas_to_mz(formulas)
          abundances, s = get_abundances(formulas, experiments, mz, method=method,␣
↪detection_limit=detection_limit, mz_tol=mz_tol)
          return normalized(abundances), s
```

Make a scatter plot of the abundances.

```
[12]: def plot_scatter(abundances, formulas):
          plt.gca().set_prop_cycle(None)
          for formula in sorted(formulas):
              plt.scatter(abundances['t'], abundances[formula], marker=ms('o',␣
↪fillstyle='none'), label=format_formula(formula))
          plt.legend()
          plt.xlabel(r'$t/\mathrm{s}$')
          plt.ylabel('$I$')
```

Calculate the total rate constant while preserving correlation.

```
[13]: def calc_k_tot(fit_result, Z=1):
          ks = list(map(unc.ufloat, fit_result.params.values(), Z*np.sqrt(np.diag(fit_result.
↪covariance_matrix))))
          return ks, np.sum(ks)
```

Apply a fix in spike for loading of unprocessed datasets.

```
[14]: from spike.File import BrukerNMR as bkn

      def read_param(filename="acqus"):
          if filename is None:
              return

          return bkn.read_param(filename=filename, get_title=False)
```

```
bkn.find_proc = lambda x: None
spike.File.Apex0.read_param = read_param
```

Solve model differential equation.

```
[15]: def solve_model(eqs, funcs, ics, check=True):
          sols = sf.dsolve(eqs, funcs, ics=ics)

          # Sanity check
          if check:
              assert sum(list(map(lambda sol: sol.rhs, sols))).simplify() == 1, 'Solutions␣
       ↪do not sum to 1.'

          return sf.Model({sf.symbols(sol.lhs.name): sol.rhs for sol in sols})
```

Fit solved model to data.

```
[16]: def fit_model(model, abundances, sigmas, ignore, verbose=False, **kwargs):
          data = {key.replace('+',''): np.nan_to_num(abundances[key]) if key != ignore else␣
       ↪None for key in abundances.dtype.fields}
          unc = {'sigma_' + key.replace('+',''): np.nan_to_num(sigmas[key]) for key in␣
       ↪sigmas.dtype.fields if key != 't'}
          fit = sf.Fit(model, **data, **unc, absolute_sigma=False, **kwargs)
          fit_result = fit.execute()
          if verbose:
              print(fit_result)

          return fit_result
```

Plot fitted model.

```
[17]: def plot_fit(T, fit_result):
          res = fit_result.model(t=T, **fit_result.params)
          plt.gca().set_prop_cycle(None)
          for curve in res:
              plt.plot(T, curve)
```

Calculate propagated uncertainty.

```
[18]: def calc_unc(result):
          J = sf.Matrix(result.model.jacobian)
          C = result.covariance_matrix
          Cp = J*C*J.T
          return sf.Matrix(np.diag(Cp)).T
```

Plot uncertainty bands.

```
[19]: def plot_unc(T, results, Z=1, **kwargs):
          res = results.model(t=T, **results.params)
          for curve, err in zip(res, calc_unc(results)):
              s = Z*np.sqrt(err(t=T, **results.params))
              plt.fill_between(T, curve + s, curve - s, alpha=0.5)
```

Process data for $C_2H_5^+ + C_6H_6$

[20]:
```
ethanium_formulas = ['C6H7+', 'C2H5+']
ethanium_abundances, ethanium_s = get_normalized_abundances('./20240219_C2H5+_C6H6_2e-8mBar_3000ms/', ethanium_formulas)

k = sf.Parameter('k')
t, = sf.variables('t')
T = np.linspace(0, 3)

C2H5 = sf.Function('C2H5')
C6H7 = sf.Function('C6H7')

eqs = [
       sf.Eq(C2H5(t).diff(t), -k*C2H5(t)),
       sf.Eq(C6H7(t).diff(t),  k*C2H5(t)),
      ]

funcs = [
    C2H5(t),
    C6H7(t)
]
ics = {
    C2H5(0): 1,
    C6H7(0): 0
}

model = solve_model(eqs, funcs, ics, check=True)
fit_ethanium_result = fit_model(model, ethanium_abundances, ethanium_s, ignore='C2H5+')

plt.figure()
plot_scatter(ethanium_abundances, ethanium_formulas)
plt.ylabel(r'$I/\mathrm{a.u.}$')
plt.xlabel(r'$t/\mathrm{s}$')
plot_fit(T, fit_ethanium_result)
plot_unc(T, fit_ethanium_result)
plt.show()

k_ethanium_lit = 3.0e-9 # cm^3 / molecule / s
k_ethanium_obs, = unc.correlated_values(fit_ethanium_result.params.values(),␣
 ↪fit_ethanium_result.covariance_matrix)
nd_ethanium = k_ethanium_obs / k_ethanium_lit
print('nd = {:.1u} molecule / cm^3'.format(nd_ethanium))
kB = 1.380649e-23 # J/K
temp = 298 # K
p_ethanium = nd_ethanium * kB * temp * 1e4
print('P  = {:.1u} mBar'.format(p_ethanium))
```

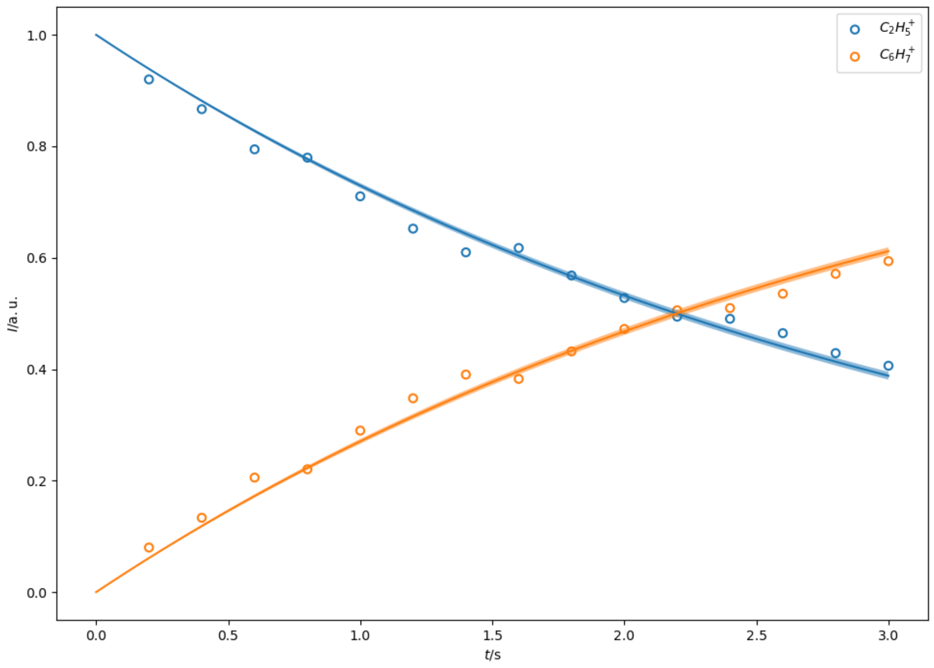


```
nd = (1.05+/-0.02)e+08 molecule / cm^3
P  = (4.33+/-0.08)e-09 mBar
```

Process data for $H_3O^+ + C_6H_6$

[21]:
```
hydronium_formulas = ['C6H7+', 'H3O+']
hydronium_abundances, hydronium_s = get_normalized_abundances('./20240214_H3O+_C6H6_2e-8mBar_3000ms/', hydronium_formulas)

H3O = sf.Function('H3O')
C6H7 = sf.Function('C6H7')

eqs = [
       sf.Eq(H3O(t).diff(t), -k*H3O(t)),
       sf.Eq(C6H7(t).diff(t),  k*H3O(t)),
      ]

funcs = [
    H3O(t),
    C6H7(t)
]
ics = {
    H3O(0): 1,
    C6H7(0): 0
```

```
}

model = solve_model(eqs, funcs, ics, check=True)
fit_hydronium_result = fit_model(model, hydronium_abundances, hydronium_s,␣
 ↪ignore='H3O+')

plt.figure()
plot_scatter(hydronium_abundances, hydronium_formulas)
plt.ylabel(r'$I/\mathrm{a.u.}$')
plt.xlabel(r'$t/\mathrm{s}$')
plot_fit(T, fit_hydronium_result)
plot_unc(T, fit_hydronium_result)
plt.show()

k_hydronium_lit = 1.9e-9 # cm^3 / molecule / s
k_hydronium_obs, = unc.correlated_values(fit_hydronium_result.params.values(),␣
 ↪fit_hydronium_result.covariance_matrix)
nd_hydronium = k_hydronium_obs / k_hydronium_lit
print('nd = {:.1u} molecule / cm^3'.format(nd_hydronium))
p_hydronium = nd_hydronium * kB * temp * 1e4
print('P  = {:.1u} mBar'.format(p_hydronium))
```

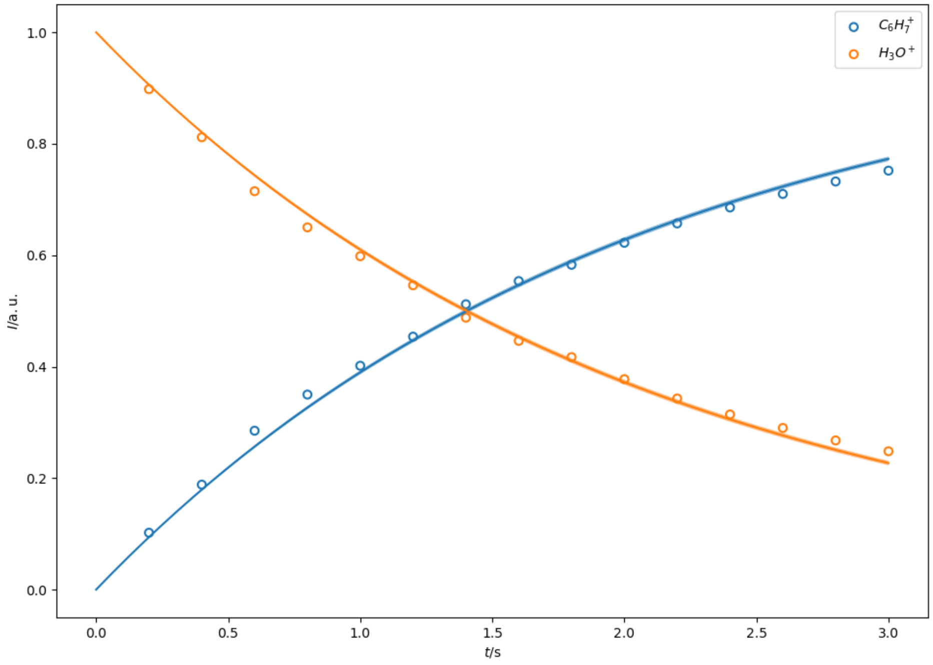


```
nd = (2.60+/-0.03)e+08 molecule / cm^3
P  = (1.07+/-0.01)e-08 mBar
```

Process data for $H_3O_2^+ + C_6H_6$

[22]:
```
hydroperoxonium_formulas = ['C6H6+', 'C6H5+', 'C6H7+', 'H3O2+']
hydroperoxonium_abundances, hydroperoxonium_s = get_normalized_abundances('./
↪20231120_H3O2+_C6H6_2e-8mBar_3000ms/', hydroperoxonium_formulas, detection_limit=3)
hydroperoxonium_abundances['C6H6+'] -= 0.066 * hydroperoxonium_abundances['C6H5+']
hydroperoxonium_abundances['C6H7+'] -=  0.066 * hydroperoxonium_abundances['C6H6+'] +␣
↪0.002 * hydroperoxonium_abundances['C6H5+']

tiny = np.finfo(np.float64).eps
k_pt = sf.Parameter('k_pt', min=tiny)
k_et = sf.Parameter('k_et', min=tiny)
k_ht = sf.Parameter('k_ht', min=tiny)
t, = sf.variables('t')
T = np.linspace(0, 3)

H3O2 = sf.Function('H3O2')
C6H5 = sf.Function('C6H5')
C6H6 = sf.Function('C6H6')
C6H7 = sf.Function('C6H7')

eqs = [
       sf.Eq(H3O2(t).diff(t), -k_ht*H3O2(t) - k_et*H3O2(t) - k_pt*H3O2(t)),
       sf.Eq(C6H5(t).diff(t),  k_ht*H3O2(t)),
       sf.Eq(C6H6(t).diff(t),  k_et*H3O2(t)),
       sf.Eq(C6H7(t).diff(t),  k_pt*H3O2(t))
      ]
funcs = [
    H3O2(t),
    C6H5(t),
    C6H6(t),
    C6H7(t)
]
ics = {
    H3O2(0): 1,
    C6H5(0): 0,
    C6H6(0): 0,
    C6H7(0): 0
}

model = solve_model(eqs, funcs, ics, check=True)
fit_result = fit_model(model, hydroperoxonium_abundances, hydroperoxonium_s,␣
↪ignore='H3O2+')

plt.figure()
plot_scatter(hydroperoxonium_abundances, hydroperoxonium_formulas)
plt.ylabel(r'$I/\mathrm{a.u.}$')
plt.xlabel(r'$t/\mathrm{s}$')
plot_fit(T, fit_result)
plot_unc(T, fit_result)
plt.show()
```

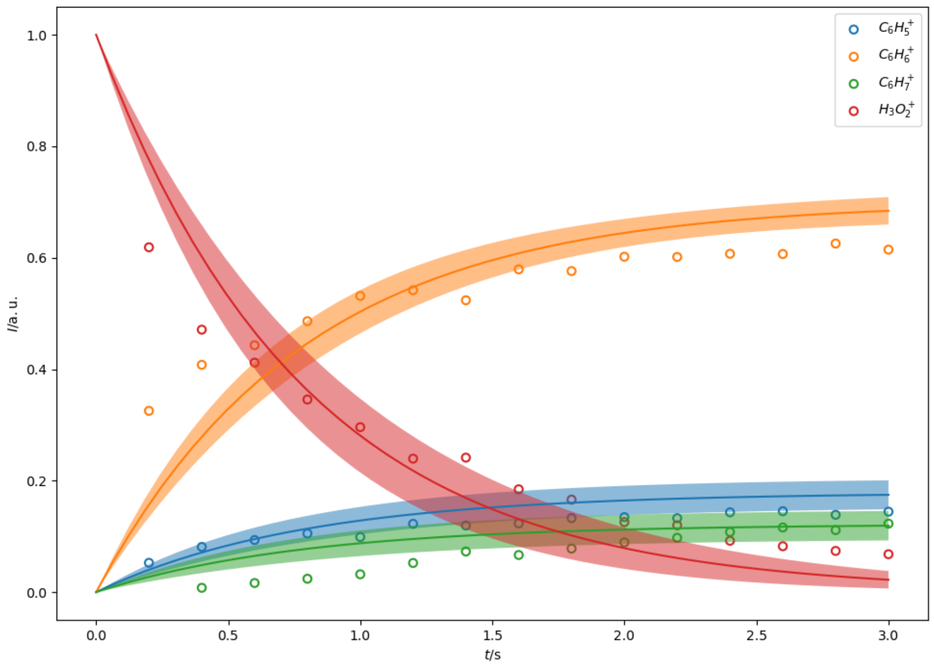

Use the more certain H_3O^+-derived pressure, but take the uncertainty to be the difference between the pressure deviation between the two methods.

[23]:
```
nd = unc.ufloat(nd_hydronium.n, np.abs(nd_hydronium.n - nd_ethanium.n))
print('nd = {:.1u} molecule / cm^3'.format(nd))
p = nd * kB * temp * 1e4
print('P  = {:.1u} mBar'.format(p))
```

```
nd = (3+/-2)e+08 molecule / cm^3
P  = (1.1+/-0.6)e-08 mBar
```

Calculate Langvin capture rate for C6D6.

[24]:
```
alpha = 10.33 * 1e-30           # m^3, benzene polarizability volume
q     = 1 * 1.60e-19            # C, charge
eps0  = 8.854e-12               # F/m, vacuum permittivity
m1    = 78.04695 * 1.66054E-27  # kg, benzene monoisotopic mass
m2    = 35.01276 * 1.66054E-27  # kg, H3O2+ monoisotopic mass
mu    = m1 * m2 / (m1 + m2)     # kg, reduced mass

kL = q * np.sqrt(np.pi*alpha/mu/eps0) # m^3/s, Langevin rate constant
kL *= 1e6                             # cm^3/s, Langevin rate constant
```

Print all the rate constants.

[25]:
```
ks, k_tot = calc_k_tot(fit_result)

for k_name, k in zip(fit_result.params.keys(), ks):
    print('{} = {:.2u} cm^3 / s'.format(k_name, k / nd))
print('-'*32)
print('k_tot = {:2.2u} cm^3 / s'.format(k_tot / nd))
print('='*32)
print('k_L = {:.2g} cm^3 / s'.format(kL))
```

```
k_et = (3.4+/-2.1)e-09 cm^3 / s
k_ht = (8.7+/-5.7)e-10 cm^3 / s
k_pt = (6.0+/-4.1)e-10 cm^3 / s
--------------------------------
k_tot = 4.9e-09+/-3.0e-09 cm^3 / s
================================
k_L = 1.5e-09 cm^3 / s
```

Print the branching ratios.

[26]:
```
for k_name, k in zip(fit_result.params.keys(), ks):
    print('{} = {:.1u} %'.format(k_name, k / k_tot * 100))
```

```
k_et = 70+/-6 %
k_ht = 18+/-4 %
k_pt = 12+/-4 %
```

Process data for $H_3O_2^+ + C_6D_6$

[27]:
```
hydroperoxonium_D_formulas = ['C6D6H+', 'C6D6+', 'C6D5+', 'C6D4H+', 'H3O2+']
hydroperoxonium_D_abundances, hydroperoxonium_D_s = get_normalized_abundances('./
 ↪20231221_H3O2+_C6D6_2e-8mBar_3000ms/', hydroperoxonium_D_formulas, detection_limit=3)
hydroperoxonium_D_abundances['C6D5+'] -= 0.065 * np.
 ↪nan_to_num(hydroperoxonium_D_abundances['C6D4H+'])
hydroperoxonium_D_abundances['C6D6+'] -= 0.002 * np.
 ↪nan_to_num(hydroperoxonium_D_abundances['C6D5+'])
hydroperoxonium_D_abundances['C6D6H+'] -= 0.065 * np.
 ↪nan_to_num(hydroperoxonium_D_abundances['C6D6+'])

k_pt_D = sf.Parameter('k_pt_D', min=tiny)
k_et_D = sf.Parameter('k_et_D', min=tiny)
k_ht_D = sf.Parameter('k_ht_D', min=tiny)
k_ae_D = sf.Parameter('k_ae_D', min=tiny)
t, = sf.variables('t')
T = np.linspace(0, 3)

H3O2  = sf.Function('H3O2')
C6D4H = sf.Function('C6D4H')
C6D5  = sf.Function('C6D5')
C6D6  = sf.Function('C6D6')
C6D6H = sf.Function('C6D6H')

eqs = [
```

```python
        sf.Eq(H3O2(t).diff(t),  -k_ht_D*H3O2(t) - k_et_D*H3O2(t) - k_pt_D*H3O2(t)  -␣
↪k_ae_D*H3O2(t)),
        sf.Eq(C6D4H(t).diff(t),  k_ae_D*H3O2(t)),
        sf.Eq(C6D5(t).diff(t),   k_ht_D*H3O2(t)),
        sf.Eq(C6D6(t).diff(t),   k_et_D*H3O2(t)),
        sf.Eq(C6D6H(t).diff(t),  k_pt_D*H3O2(t))
      ]
funcs = [
    H3O2(t),
    C6D4H(t),
    C6D5(t),
    C6D6(t),
    C6D6H(t)
]
ics = {
    H3O2(0):  1,
    C6D4H(0): 0,
    C6D5(0):  0,
    C6D6(0):  0,
    C6D6H(0): 0
}

model = solve_model(eqs, funcs, ics, check=True)
fit_result = fit_model(model, hydroperoxonium_D_abundances, hydroperoxonium_D_s,␣
↪ignore='H3O2+')

plt.figure()
plot_scatter(hydroperoxonium_D_abundances, hydroperoxonium_D_formulas)
plt.ylabel(r'$I/\mathrm{a.u.}$')
plt.xlabel(r'$t/\mathrm{s}$')
plot_fit(T, fit_result)
plot_unc(T, fit_result)
plt.show()
```

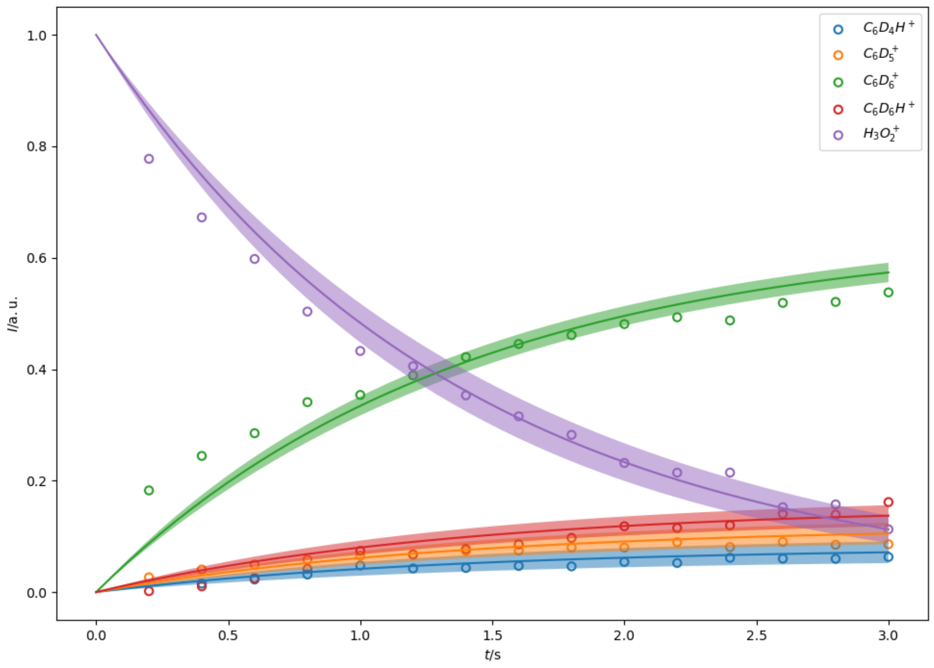

Calculate Langvin capture rate for C6D6.

[28]:
```
m1_D   = 84.08461 * 1.66054E-27 # kg, benzene monoisotopic mass
mu_D   = m1 * m2 / (m1 + m2)     # kg, reduced mass

kL_D = q * np.sqrt(np.pi*alpha/mu/eps0) # m^3/s, Langevin rate constant
kL_D *= 1e6                             # cm^3/s, Langevin rate constant
```

Print all the rate constants.

[29]:
```
ks_D, k_tot_D = calc_k_tot(fit_result)

for k_name, k in zip(fit_result.params.keys(), ks_D):
    print('{} = {:.2u} cm^3 / s'.format(k_name, k / nd))
print('-'*32)
print('k_tot = {:2.2u} cm^3 / s'.format(k_tot_D / nd))
print('='*32)
print('k_L_D = {:.2g} cm^3 / s'.format(kL_D))
```

```
k_ae_D = (2.3+/-1.5)e-10 cm^3 / s
k_et_D = (1.8+/-1.1)e-09 cm^3 / s
k_ht_D = (3.3+/-2.1)e-10 cm^3 / s
k_pt_D = (4.3+/-2.7)e-10 cm^3 / s
--------------------------------
k_tot = 2.8e-09+/-1.7e-09 cm^3 / s
```

```
=================================
k_L_D = 1.5e-09 cm^3 / s
```

Print the branching ratios.

[30]:
```
for k_name, k in zip(fit_result.params.keys(), ks_D):
    print('{} = {:.1u} %'.format(k_name, k / k_tot_D * 100))
```

```
k_ae_D = 8+/-2 %
k_et_D = 65+/-3 %
k_ht_D = 12+/-2 %
k_pt_D = 15+/-3 %
```

Assuming complete scrambling of hydrogens and deuteriums in the electrophilic addition-elimination mechanism, calculate the corrected rate constants and branching ratios.

[31]:
```
k_ae_D, k_et_D, k_ht_D, k_pt_D = ks_D

k_ae_D_adj = k_ae_D + k_ae_D * 2 / 7
k_ht_D_adj = k_ht_D - k_ae_D * 2 / 7
print('k_ae_D_adj = {:.1g} cm^3 / s'.format(k_ae_D_adj / nd))
print('k_ht_D_adj = {:.1g} cm^3 / s'.format(k_ht_D_adj / nd))
print('-'*33)
print('k_ae_D_adj = {:.1u} %'.format(k_ae_D_adj / k_tot_D * 100))
print('k_ht_D_adj = {:.1u} %'.format(k_ht_D_adj / k_tot_D * 100))
```

```
k_ae_D_adj = (3+/-2)e-10 cm^3 / s
k_ht_D_adj = (3+/-2)e-10 cm^3 / s
---------------------------------
k_ae_D_adj = 10+/-3 %
k_ht_D_adj = 10+/-3 %
```

Alternative models

Non-zero initial intensities

[32]:
```
r0 = sf.Parameter('r0')
pt0 = sf.Parameter('pt0')
et0 = sf.Parameter('et0')
ht0 = sf.Parameter('ht0')

eqs = [
       sf.Eq(H3O2(t).diff(t), -k_ht*H3O2(t) - k_et*H3O2(t) - k_pt*H3O2(t)),
       sf.Eq(C6H5(t).diff(t),  k_ht*H3O2(t)),
       sf.Eq(C6H6(t).diff(t),  k_et*H3O2(t)),
       sf.Eq(C6H7(t).diff(t),  k_pt*H3O2(t))
      ]
funcs = [
    H3O2(t),
    C6H5(t),
    C6H6(t),
    C6H7(t)
```

```python
]
ics = {
    H3O2(0): r0,
    C6H5(0): ht0,
    C6H6(0): et0,
    C6H7(0): pt0
}

model = solve_model(eqs, funcs, ics, check=False)
fit_result = fit_model(model, hydroperoxonium_abundances, hydroperoxonium_s,␣
↪ignore='', verbose=True)

plt.figure()
plot_scatter(hydroperoxonium_abundances, hydroperoxonium_formulas)
plt.ylabel(r'$I/\mathrm{a.u.}$')
plt.xlabel(r'$t/\mathrm{s}$')
plot_fit(T, fit_result)
plot_unc(T, fit_result)
plt.show()
```

```
Parameter Value        Standard Deviation
et0       2.934428e-01 2.419218e-02
ht0       4.000722e-02 2.269258e-02
k_et      4.448482e-01 4.173690e-02
k_ht      1.414978e-01 3.806801e-02
k_pt      2.431318e-01 4.430447e-02
pt0       -7.005325e-02 2.747937e-02
r0        6.899198e-01 2.903866e-02
Status message         CONVERGENCE: REL_REDUCTION_OF_F_<=_FACTR*EPSMCH
Number of iterations   27
Objective              <symfit.core.objectives.LeastSquares object at
0x0000027BED703B10>
Minimizer              <symfit.core.minimizers.LBFGSB object at
0x0000027BEE89EBD0>

Goodness of fit qualifiers:
chi_squared            0.008148547270276607
objective_value        0.004074273635138304
r_squared              0.9815027921162311
```

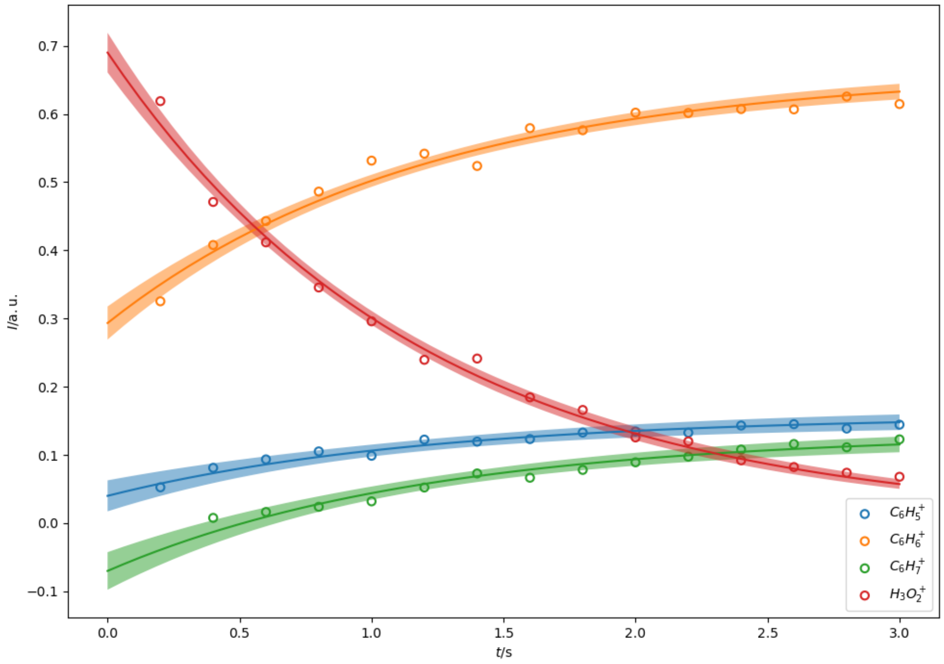

Non-zero initial intensities summing to 1

[33]:

```
pt0 = sf.Parameter('pt0')
et0 = sf.Parameter('et0')
ht0 = sf.Parameter('ht0')

eqs = [
       sf.Eq(H3O2(t).diff(t), -k_ht*H3O2(t) - k_et*H3O2(t) - k_pt*H3O2(t)),
       sf.Eq(C6H5(t).diff(t),  k_ht*H3O2(t)),
       sf.Eq(C6H6(t).diff(t),  k_et*H3O2(t)),
       sf.Eq(C6H7(t).diff(t),  k_pt*H3O2(t))
      ]
funcs = [
    H3O2(t),
    C6H5(t),
    C6H6(t),
    C6H7(t)
]
ics = {
    H3O2(0): 1 - ht0 - et0 - pt0,
    C6H5(0): ht0,
    C6H6(0): et0,
    C6H7(0): pt0
}
```

```
model = solve_model(eqs, funcs, ics, check=False)
fit_result = fit_model(model, hydroperoxonium_abundances, hydroperoxonium_s,␣
↪ignore='H3O2+', verbose=True)

plt.figure()
plot_scatter(hydroperoxonium_abundances, hydroperoxonium_formulas)
plt.ylabel(r'$I/\mathrm{a.u.}$')
plt.xlabel(r'$t/\mathrm{s}$')
plot_fit(T, fit_result)
plot_unc(T, fit_result)
plt.show()
```

```
Parameter Value        Standard Deviation
et0       3.134205e-01 2.302753e-02
ht0       4.719260e-02 2.005374e-02
k_et      3.665954e-01 3.494685e-02
k_ht      1.163362e-01 2.981873e-02
k_pt      1.967051e-01 3.646111e-02
pt0       -5.585087e-02 2.613922e-02
Status message         CONVERGENCE: REL_REDUCTION_OF_F_<=_FACTR*EPSMCH
Number of iterations   32
Objective              <symfit.core.objectives.LeastSquares object at
0x0000027BEC4DE190>
Minimizer              <symfit.core.minimizers.LBFGSB object at
0x0000027BEEA04550>

Goodness of fit qualifiers:
chi_squared            0.006182137966915499
objective_value        0.0030910689834577495
r_squared              0.9503746641271813
```

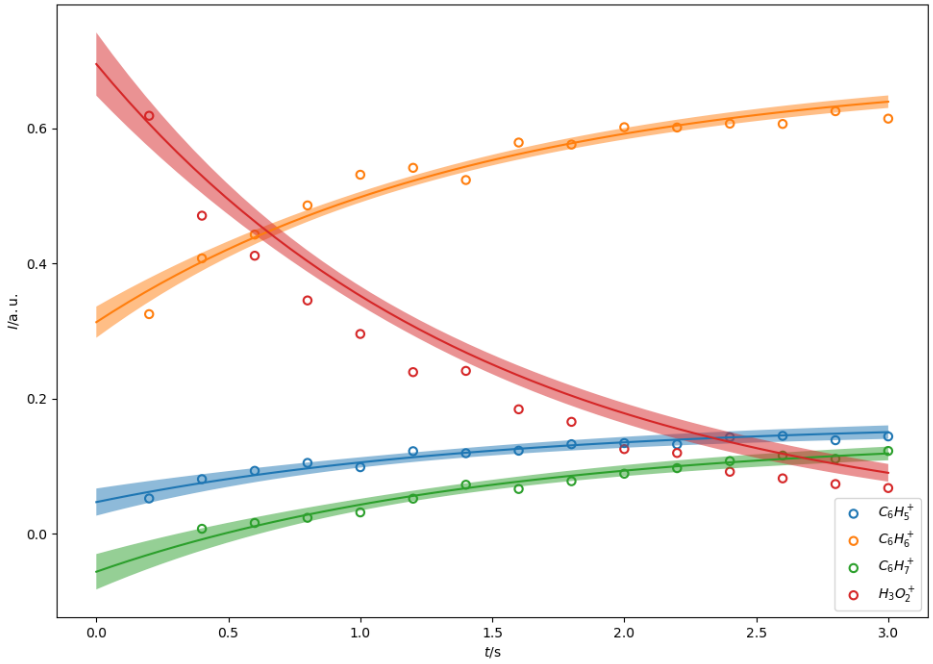

Positive initial intensities

[34]:
```
r0 = sf.Parameter('r0', min=0)
pt0 = sf.Parameter('pt0', min=0)
et0 = sf.Parameter('et0', min=0)
ht0 = sf.Parameter('ht0', min=0)

eqs = [
       sf.Eq(H3O2(t).diff(t), -k_ht*H3O2(t) - k_et*H3O2(t) - k_pt*H3O2(t)),
       sf.Eq(C6H5(t).diff(t),  k_ht*H3O2(t)),
       sf.Eq(C6H6(t).diff(t),  k_et*H3O2(t)),
       sf.Eq(C6H7(t).diff(t),  k_pt*H3O2(t))
      ]
funcs = [
    H3O2(t),
    C6H5(t),
    C6H6(t),
    C6H7(t)
]
ics = {
    H3O2(0): r0,
    C6H5(0): ht0,
    C6H6(0): et0,
    C6H7(0): pt0
```

```python
}

model = solve_model(eqs, funcs, ics, check=False)
fit_result = fit_model(model, hydroperoxonium_abundances, hydroperoxonium_s, ignore='', verbose=True)

plt.figure()
plot_scatter(hydroperoxonium_abundances, hydroperoxonium_formulas)
plt.ylabel(r'$I/\mathrm{a.u.}$')
plt.xlabel(r'$t/\mathrm{s}$')
plot_fit(T, fit_result)
plot_unc(T, fit_result)
plt.show()
```

```
Parameter Value        Standard Deviation
et0       2.803102e-01 3.287841e-02
ht0       2.318483e-02 3.072298e-02
k_et      4.752137e-01 5.782937e-02
k_ht      1.744669e-01 5.222686e-02
k_pt      1.366502e-01 5.701443e-02
pt0       0.000000e+00 3.507709e-02
r0        6.607713e-01 3.760644e-02
Status message         CONVERGENCE: REL_REDUCTION_OF_F_<=_FACTR*EPSMCH
Number of iterations   22
Objective              <symfit.core.objectives.LeastSquares object at
0x0000027BEF3B4050>
Minimizer              <symfit.core.minimizers.LBFGSB object at
0x0000027BEF3D5610>

Goodness of fit qualifiers:
chi_squared            0.01508311421483079
objective_value        0.007541557107415395
r_squared              0.9706460374280408
```

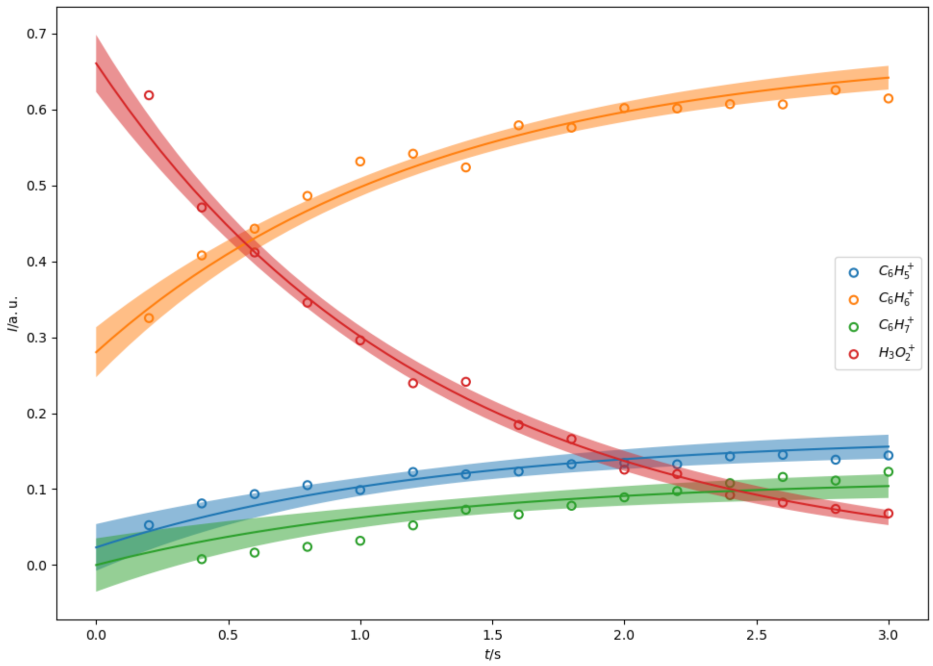

Positive initial intensities summing to 1

[35]:
```
pt0 = sf.Parameter('pt0', min=0)
et0 = sf.Parameter('et0', min=0)
ht0 = sf.Parameter('ht0', min=0)

eqs = [
       sf.Eq(H3O2(t).diff(t), -k_ht*H3O2(t) - k_et*H3O2(t) - k_pt*H3O2(t)),
       sf.Eq(C6H5(t).diff(t),  k_ht*H3O2(t)),
       sf.Eq(C6H6(t).diff(t),  k_et*H3O2(t)),
       sf.Eq(C6H7(t).diff(t),  k_pt*H3O2(t))
      ]
funcs = [
    H3O2(t),
    C6H5(t),
    C6H6(t),
    C6H7(t)
]
ics = {
    H3O2(0): 1 - ht0 - et0 - pt0,
    C6H5(0): ht0,
    C6H6(0): et0,
    C6H7(0): pt0
}
```

```
model = solve_model(eqs, funcs, ics, check=False)
fit_result = fit_model(model, hydroperoxonium_abundances, hydroperoxonium_s,␣
↪ignore='', verbose=True)

plt.figure()
plot_scatter(hydroperoxonium_abundances, hydroperoxonium_formulas)
plt.ylabel(r'$I/\mathrm{a.u.}$')
plt.xlabel(r'$t/\mathrm{s}$')
plot_fit(T, fit_result)
plot_unc(T, fit_result)
plt.show()
```

```
Parameter Value        Standard Deviation
et0       2.953793e-01 3.430610e-02
ht0       3.614352e-02 3.242588e-02
k_et      4.514919e-01 5.880698e-02
k_ht      1.630399e-01 5.428616e-02
k_pt      1.470993e-01 5.938870e-02
pt0       0.000000e+00 3.678845e-02
Status message         CONVERGENCE: REL_REDUCTION_OF_F_<=_FACTR*EPSMCH
Number of iterations   29
Objective              <symfit.core.objectives.LeastSquares object at
0x0000027BED03C1D0>
Minimizer              <symfit.core.minimizers.LBFGSB object at
0x0000027BEFFCB0D0>

Goodness of fit qualifiers:
chi_squared            0.02008659677249582
objective_value        0.01004329838624791
r_squared              0.960983245020854
```

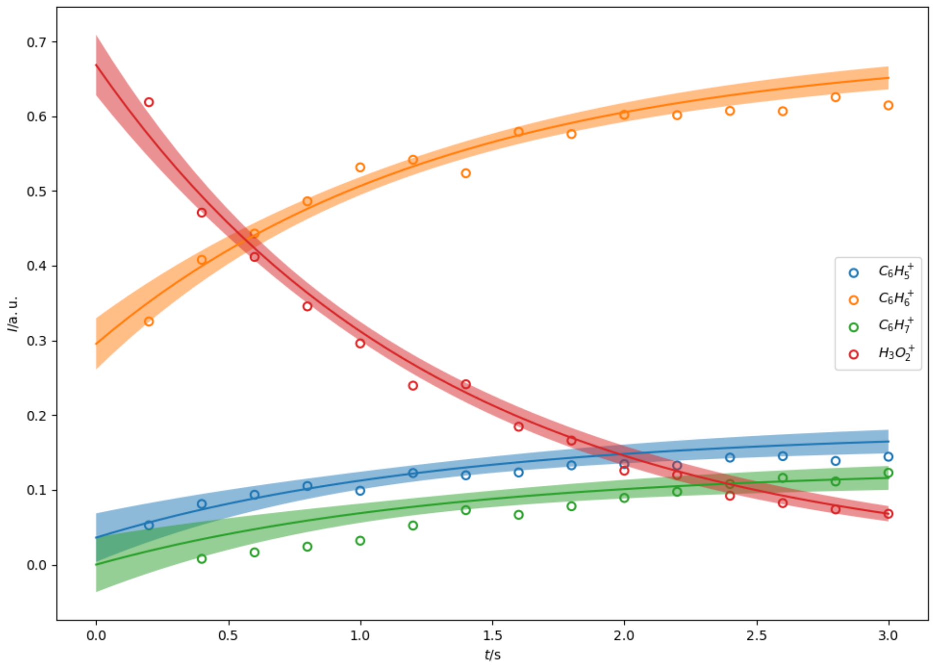

$C_6H_7^+$ as secondary product

Zero initial intensities

[36]:
```
k_secP = sf.Parameter('k_secP', min=0)

eqs = [
       sf.Eq(H3O2(t).diff(t), -k_ht*H3O2(t) - k_et*H3O2(t) - k_pt*H3O2(t)),
       sf.Eq(C6H5(t).diff(t),  k_ht*H3O2(t)),
       sf.Eq(C6H6(t).diff(t),  k_et*H3O2(t) - k_secP*C6H6(t)),
       sf.Eq(C6H7(t).diff(t),  k_pt*H3O2(t) + k_secP*C6H6(t))
      ]
funcs = [
    H3O2(t),
    C6H5(t),
    C6H6(t),
    C6H7(t)
]
ics = {
    H3O2(0): 1,
    C6H5(0): 0,
    C6H6(0): 0,
    C6H7(0): 0
}
```

```
model = solve_model(eqs, funcs, ics, check=False)
fit_result = fit_model(model, hydroperoxonium_abundances, hydroperoxonium_s,␣
↪ignore='', verbose=True)

plt.figure()
plot_scatter(hydroperoxonium_abundances, hydroperoxonium_formulas)
plt.ylabel(r'$I/\mathrm{a.u.}$')
plt.xlabel(r'$t/\mathrm{s}$')
plot_fit(T, fit_result)
plot_unc(T, fit_result)
plt.show()
```

```
Parameter Value        Standard Deviation
k_et      9.922022e-01 1.188783e-01
k_ht      2.161509e-01 4.722307e-02
k_pt      1.222287e-02 1.001846e-01
k_secP    1.069524e-01 6.892999e-02
Status message         CONVERGENCE: REL_REDUCTION_OF_F_<=_FACTR*EPSMCH
Number of iterations   15
Objective              <symfit.core.objectives.LeastSquares object at
0x0000027BEFAF8310>
Minimizer              <symfit.core.minimizers.LBFGSB object at
0x0000027BEFA9D690>

Goodness of fit qualifiers:
chi_squared            0.14417729995306866
objective_value        0.07208864997653433
r_squared              0.7244776487694569
```

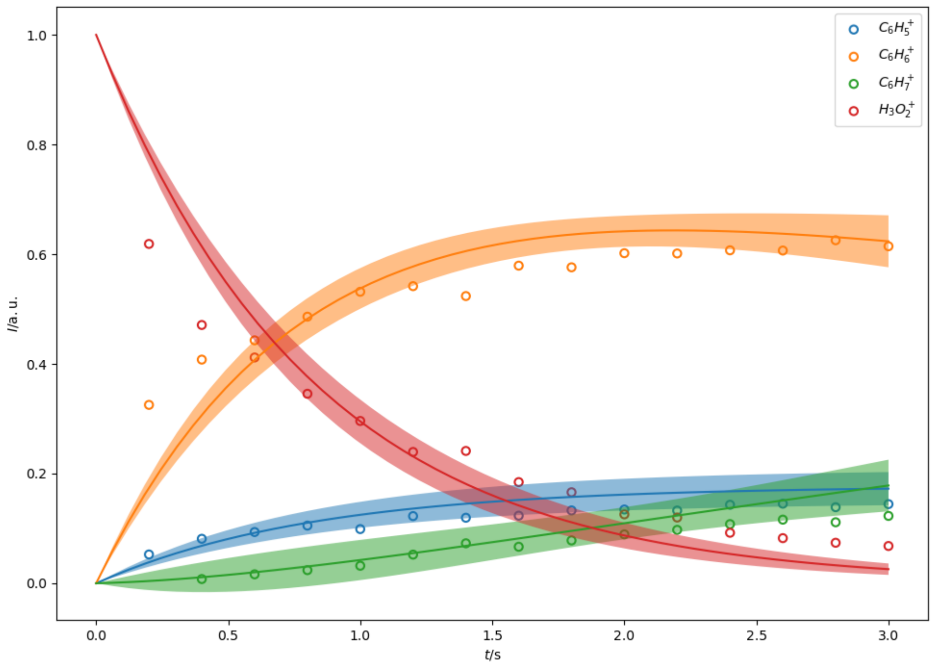

Positive initial intensities

[37]:
```
k_secP = sf.Parameter('k_secP', min=0)

eqs = [
       sf.Eq(H3O2(t).diff(t), -k_ht*H3O2(t) - k_et*H3O2(t) - k_pt*H3O2(t)),
       sf.Eq(C6H5(t).diff(t),  k_ht*H3O2(t)),
       sf.Eq(C6H6(t).diff(t),  k_et*H3O2(t) - k_secP*C6H6(t)),
       sf.Eq(C6H7(t).diff(t),  k_pt*H3O2(t) + k_secP*C6H6(t))
      ]
funcs = [
    H3O2(t),
    C6H5(t),
    C6H6(t),
    C6H7(t)
]
ics = {
    H3O2(0): r0,
    C6H5(0): ht0,
    C6H6(0): et0,
    C6H7(0): pt0
}

model = solve_model(eqs, funcs, ics, check=False)
```

```python
fit_result = fit_model(model, hydroperoxonium_abundances, hydroperoxonium_s,␣
↪ignore='', verbose=True)

plt.figure()
plot_scatter(hydroperoxonium_abundances, hydroperoxonium_formulas)
plt.ylabel(r'$I/\mathrm{a.u.}$')
plt.xlabel(r'$t/\mathrm{s}$')
plot_fit(T, fit_result)
plot_unc(T, fit_result)
plt.show()
```

```
Parameter Value        Standard Deviation
et0       2.518874e-01 3.297789e-02
ht0       3.332342e-02 2.358606e-02
k_et      6.254965e-01 1.038969e-01
k_ht      1.545382e-01 3.976525e-02
k_pt      2.344165e-02 1.144092e-01
k_secP    7.312550e-02 4.045450e-02
pt0       0.000000e+00 3.914976e-02
r0        6.739452e-01 2.983782e-02
Status message         CONVERGENCE: REL_REDUCTION_OF_F_<=_FACTR*EPSMCH
Number of iterations   34
Objective              <symfit.core.objectives.LeastSquares object at
0x0000027BEFB81C90>
Minimizer              <symfit.core.minimizers.LBFGSB object at
0x0000027BEEB284D0>

Goodness of fit qualifiers:
chi_squared            0.0077799757207557985
objective_value        0.0038899878603778993
r_squared              0.9850363392488064
```

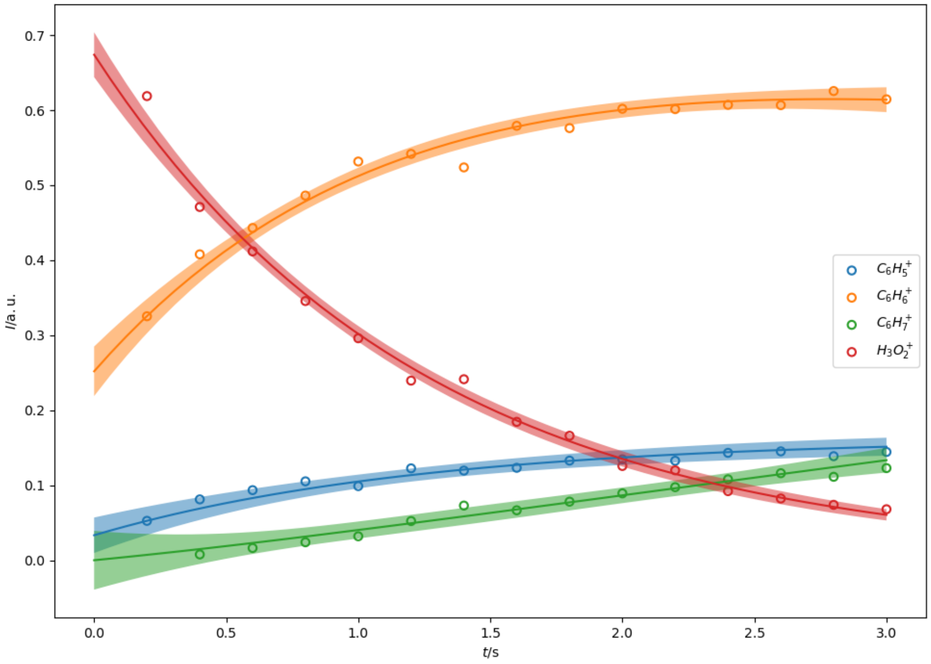

Non-zero initial intensities

[38]:
```
pt0 = sf.Parameter('pt0')

eqs = [
       sf.Eq(H3O2(t).diff(t), -k_ht*H3O2(t) - k_et*H3O2(t) - k_pt*H3O2(t)),
       sf.Eq(C6H5(t).diff(t),  k_ht*H3O2(t)),
       sf.Eq(C6H6(t).diff(t),  k_et*H3O2(t) - k_secP*C6H6(t)),
       sf.Eq(C6H7(t).diff(t),  k_pt*H3O2(t) + k_secP*C6H6(t))
      ]
funcs = [
    H3O2(t),
    C6H5(t),
    C6H6(t),
    C6H7(t)
]
ics = {
    H3O2(0): r0,
    C6H5(0): ht0,
    C6H6(0): et0,
    C6H7(0): pt0
}

model = solve_model(eqs, funcs, ics, check=False)
```

```
fit_result = fit_model(model, hydroperoxonium_abundances, hydroperoxonium_s,␣
↪ignore='', verbose=True)

plt.figure()
plot_scatter(hydroperoxonium_abundances, hydroperoxonium_formulas)
plt.ylabel(r'$I/\mathrm{a.u.}$')
plt.xlabel(r'$t/\mathrm{s}$')
plot_fit(T, fit_result)
plot_unc(T, fit_result)
plt.show()
```

```
Parameter Value         Standard Deviation
et0       2.700252e-01 3.017596e-02
ht0       3.876547e-02 2.190928e-02
k_et      5.506445e-01 9.401258e-02
k_ht      1.439262e-01 3.679178e-02
k_pt      1.269797e-01 1.037022e-01
k_secP    4.493615e-02 3.636352e-02
pt0       -3.926198e-02 3.636539e-02
r0        6.854288e-01 2.814737e-02
Status message         CONVERGENCE: REL_REDUCTION_OF_F_<=_FACTR*EPSMCH
Number of iterations   48
Objective              <symfit.core.objectives.LeastSquares object at
0x0000027BF1F33FD0>
Minimizer              <symfit.core.minimizers.LBFGSB object at
0x0000027BF1EFBB50>

Goodness of fit qualifiers:
chi_squared            0.006666314510084371
objective_value        0.0033331572550421855
r_squared              0.9864612669799596
```

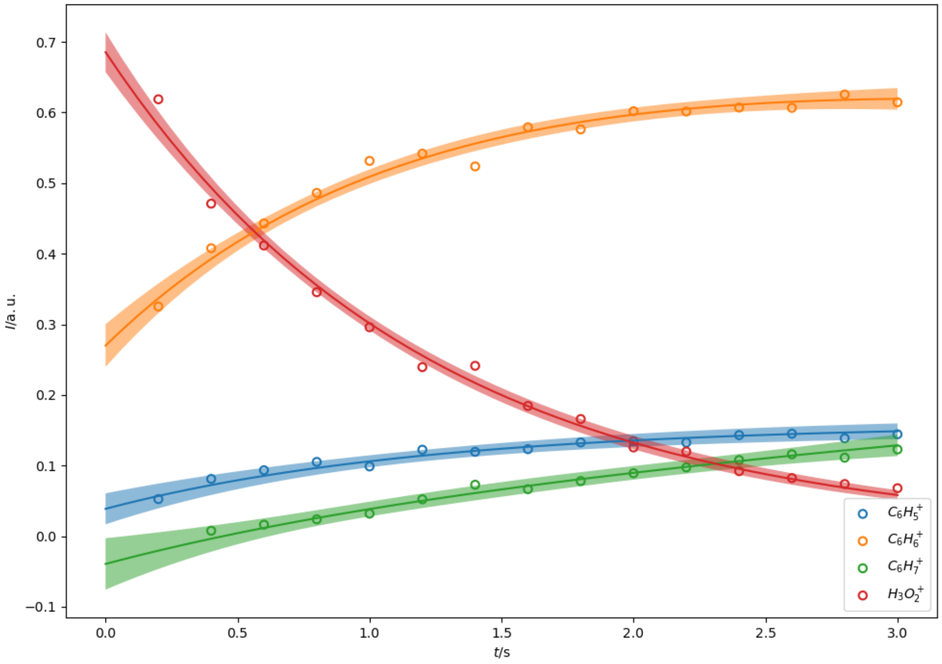
0.7
0.6
0.5
0.4
0.3
0.2
0.1
0.0
−0.1
I/a.u.
0.0
0.5
1.0
1.5
2.0
2.5
3.0
t/s
$C_6H_5^+$
$C_6H_6^+$
$C_6H_7^+$
$H_3O_2^+$

S5 Calculations

H^-

$E_{El} = -0.535\,667\,E_h$
$E_{El+ZPE} = -0.535\,667\,E_h$

Atom	x	y	z
H	0.0000	0.0000	0.0000

H_2

$E_{El} = -1.180\,023\,872\,07\,E_h$
$E_{El+ZPE} = -1.169\,960\,E_h$

Atom	x	y	z
H	0.0000	0.0000	0.3714
H	0.0000	0.0000	−0.3714

$HO^{\bullet}$

$E_{El} = -75.768\,598\,667\,1\,E_h$
$E_{El+ZPE} = -75.760\,185\,E_h$

Atom	x	y	z
O	0.0000	0.0000	0.1051
H	0.0000	0.0000	−0.8702

H_2O

$E_{El} = -76.466\,198\,035\,6\,E_h$
$E_{El+ZPE} = -76.444\,977\,E_h$

Atom	x	y	z
O	0.0000	0.1151	0.0000
H	0.7638	−0.4701	0.0000
H	−0.7638	−0.4701	0.0000

$H_2O^{\bullet+}$

$E_{El} = -76.000\,300\,927\,8\,E_h$
$E_{El+ZPE} = -75.981\,803\,E_h$

Atom	x	y	z
O	0.0000	0.0000	0.1126
H	0.0000	0.8235	−0.4659
H	0.0000	−0.8235	−0.4659

H_3O^+

$E_{El} = -76.738\,518\,890\,3\,E_h$
$E_{El+ZPE} = -76.704\,310\,E_h$

Atom	x	y	z
O	0.0000	0.0000	0.0580
H	0.0000	0.9425	−0.2116
H	−0.8162	−0.4713	−0.2116
H	0.8162	−0.4713	−0.2116

H_2O_2

$E_{El} = -151.617\,951\,104\,E_h$
$E_{El+ZPE} = -151.591\,548\,E_h$

Atom	x	y	z
O	0.0128	0.7257	−0.0679
H	0.8104	0.8913	0.4524
O	−0.0128	−0.7257	−0.0679
H	−0.8104	−0.8913	0.4524

$H_3O_2^+$

$E_{El} = -151.882\,014\,104\,E_h$
$E_{El+ZPE} = -151.842\,771\,E_h$

Atom	x	y	z
O	0.7704	0.0005	−0.1379
H	1.1135	−0.0050	0.7821
O	−0.6630	−0.0007	0.1176
H	−1.0081	−0.8086	−0.3310
H	−1.0076	0.8124	−0.3216

H_2O–$HO^{\bullet}$

$E_{El} = -152.243\,546\,198\,E_h$
$E_{El+ZPE} = -152.210\,673\,E_h$

Atom	x	y	z
O	−1.6336	0.0034	−0.0098
H	−0.6508	−0.0001	−0.0473
O	1.2428	−0.0021	−0.0388
H	1.7067	0.7672	0.3060
H	1.7116	−0.7692	0.3041

C_6H_6

$E_{El} = -232.335\,567\,968\,E_h$
$E_{El+ZPE} = -232.235\,219\,E_h$

Atom	x	y	z
C	−1.1875	−0.7240	0.0000
C	0.0334	−1.3903	0.0000
C	1.2208	−0.6663	0.0000
C	1.1875	0.7241	0.0000
C	−0.0333	1.3903	0.0000
C	−1.2208	0.6663	0.0000
H	−2.1112	−1.2875	0.0000
H	0.0593	−2.4720	0.0000
H	2.1706	−1.1846	0.0000
H	2.1113	1.2874	0.0000
H	−0.0594	2.4720	0.0000
H	−2.1706	1.1847	0.0000

$C_6H_5^{+}$

$E_{El} = -231.342\,321\,340\,E_h$
$E_{El+ZPE} = -231.257\,638\,E_h$

Atom	x	y	z
C	−1.2096	0.6134	0.0000
C	−1.2660	−0.8164	0.0000
C	0.0000	−1.1834	0.0000
C	1.2660	−0.8165	0.0000
C	1.2096	0.6133	0.0000
C	0.0000	1.2944	0.0000
H	−2.1670	1.1202	0.0000
H	−2.1781	−1.3931	0.0000
H	2.1780	−1.3933	0.0000
H	2.1671	1.1200	0.0000
H	0.0001	2.3749	0.0000

$C_6H_6^{\bullet +}$

$E_{El} = -232.000\,267\,435\,E_h$
$E_{El+ZPE} = -231.902\,844\,E_h$

Atom	x	y	z
C	−0.0130	1.3780	0.0000
C	−1.2507	0.6718	0.0000
C	−1.2381	−0.6931	0.0000
C	0.0130	−1.3780	0.0000
C	1.2507	−0.6718	0.0000
C	1.2381	0.6931	0.0000
H	−0.0221	2.4607	0.0000
H	−2.1784	1.2260	0.0000
H	−2.1555	−1.2643	0.0000
H	0.0221	−2.4607	0.0000
H	2.1784	−1.2260	0.0000
H	2.1555	1.2643	0.0000

$C_6H_7^+$

$E_{\mathrm{El}} = -232.635\,705\,740\,E_{\mathrm{h}}$
$E_{\mathrm{El+ZPE}} = -232.525\,489\,E_{\mathrm{h}}$

Atom	x	y	z
C	−0.7628	−1.2209	0.0000
C	0.6014	−1.2623	0.0000
C	0.6516	1.2372	0.0000
C	−0.7132	1.2504	0.0000
C	−1.4093	0.0282	0.0000
H	−1.3488	−2.1286	0.0000
H	1.1307	−2.2065	−0.0001
H	1.2181	2.1597	−0.0001
H	−1.2625	2.1808	0.0000
H	−2.4926	0.0499	0.0001
C	1.3907	−0.0278	0.0000
H	2.1023	−0.0421	−0.8459
H	2.1019	−0.0421	0.8463

$H_3O_2^+$–C_6H_6

$E_{\mathrm{El}} = -384.259\,480\,556\,E_{\mathrm{h}}$
$E_{\mathrm{El+ZPE}} = -384.120\,613\,E_{\mathrm{h}}$

Atom	x	y	z
O	−2.6124	0.5460	−0.5564
H	−3.4844	0.1307	−0.4115
O	−1.7584	−0.6245	−0.5411
H	−1.3177	−0.6184	−1.4169
H	−1.0004	−0.4694	0.2107
C	1.6531	0.4653	−0.8940
C	1.5937	−0.8962	−0.5961
C	0.9164	−1.3287	0.5407
C	0.2735	−0.3926	1.3705
C	0.3517	0.9759	1.0704
C	1.0358	1.3994	−0.0654
H	2.1947	0.7992	−1.7686
H	2.0955	−1.6129	−1.2312
H	0.8859	−2.3802	0.7927
H	−0.2017	−0.7204	2.2864
H	−0.1180	1.6971	1.7250
H	1.1003	2.4536	−0.2948

H_2O_2–H^+–C_6H_6

$E_{\mathrm{El}} = -384.259\,133\,574\,E_{\mathrm{h}}$
$E_{\mathrm{El+ZPE}} = -384.123\,553\,E_{\mathrm{h}}$

Atom	x	y	z
O	−2.7447	0.5147	−0.3801
H	−3.5998	0.1468	−0.0906
O	−1.9458	−0.6868	−0.4504
H	−1.6944	−0.7425	−1.3932
H	−0.9790	−0.5140	0.2260
C	1.8733	0.5076	−0.8198
C	1.7741	−0.8619	−0.5703
C	0.9460	−1.3178	0.4430
C	0.1702	−0.3975	1.1862
C	0.3138	0.9868	0.9476
C	1.1477	1.4300	−0.0676
H	2.5310	0.8593	−1.6032
H	2.3602	−1.5618	−1.1488
H	0.8761	−2.3747	0.6627
H	−0.3581	−0.7368	2.0705
H	−0.2421	1.6933	1.5485
H	1.2505	2.4880	−0.2620

H_2O_2–$C_6H_7^+$

$E_{El} = -384.269\,041\,986\ E_h$
$E_{El+ZPE} = -384.130\,921\ E_h$

Atom	x	y	z
C	2.2822	−0.8963	−0.2615
C	1.1301	−1.3590	0.3096
C	0.2949	0.9813	0.4699
C	1.4561	1.4222	−0.1033
C	2.4378	0.4858	−0.4634
H	3.0728	−1.5735	−0.5505
H	0.9829	−2.4169	0.4839
H	−0.4886	1.6706	0.7543
H	1.6282	2.4747	−0.2753
H	3.3559	0.8434	−0.9134
C	0.0583	−0.4416	0.6743
H	−0.3527	−0.6545	1.6726
H	−0.8539	−0.7052	0.0726
O	−2.7815	−0.7103	−0.5050
H	−3.1062	−0.6446	−1.4163
O	−3.0139	0.6283	−0.0054
H	−3.8313	0.5088	0.5021

i/o–OH^+–C_6H_6 TS

$E_{El} = -307.836\,837\,921\ E_h$
$E_{El+ZPE} = -307.721\,531\ E_h$

Atom	x	y	z
C	−0.3787	−1.4391	−0.1660
C	0.8562	−0.7379	−0.4624
C	−0.3783	1.4392	−0.1663
C	−1.4762	0.7272	0.1501
C	−1.4764	−0.7268	0.1502
H	−0.3922	−2.5161	−0.2392
H	1.6571	−1.2670	−0.9593
H	−0.3915	2.5162	−0.2397
H	−2.4051	1.2369	0.3656
H	−2.4055	−1.2362	0.3659
C	0.8564	0.7376	−0.4625
H	1.6575	1.2664	−0.9595
O	1.4781	−0.0001	0.8246
H	2.4489	−0.0003	0.7520

i/o–H^+–C_6H_6 TS

$E_{El} = E_h$
$E_{El+ZPE} = E_h$

Atom	x	y	z
C	1.220 820	0.711 413	0.006 793
C	0.037 769	1.415 445	−0.023 911
C	−1.188 307	0.714 635	−0.057 208
C	−1.179 871	−0.728 500	−0.057 313
C	0.054 332	−1.414 933	−0.024 151
C	1.229 065	−0.697 121	0.006 676
H	2.160 653	1.245 690	0.028 910
H	0.028 678	2.495 614	−0.035 234
H	−2.135 152	1.232 225	−0.133 582
H	−2.120 590	−1.257 121	−0.133 833
H	0.057 873	−2.495 133	−0.035 664
H	2.175 086	−1.220 365	0.028 698
H	−1.285 553	−0.007 710	1.031 306

i–H^+–C_6H_5OH–H_2O

$E_{El} = -384.357\,050\,912\ E_h$
$E_{El+ZPE} = -384.219\,140\ E_h$

Atom	x	y	z
C	−2.2991	−0.6462	−0.0147
C	−1.0825	−1.2803	0.0178
C	0.1513	−0.5069	0.1306
C	0.0400	0.9492	0.0051
C	−1.1952	1.5436	−0.0251
C	−2.3521	0.7519	−0.0518
H	−3.2120	−1.2233	−0.0432
H	−0.9876	−2.3582	0.0297
H	0.9551	1.5277	0.0185
H	−1.2788	2.6204	−0.0584
H	−3.3176	1.2386	−0.0993
O	1.2848	−1.1732	−0.2255
H	2.0939	−0.6210	−0.1155
H	0.0306	−0.4581	1.2879
O	3.4492	0.4401	0.0269
H	4.0685	0.3291	0.7577
H	3.9949	0.5547	−0.7601

i–H^+–C_6H_5OH

$E_{El} = -307.867\,555\,016\,E_h$
$E_{El+ZPE} = -307.753\,725\,E_h$

Atom	x	y	z
C	−1.1742	1.2343	−0.0299
C	0.1912	1.2597	0.0227
C	0.1983	−1.2426	0.0346
C	−1.1696	−1.2279	−0.0230
C	−1.8456	−0.0005	−0.0630
H	−1.7381	2.1545	−0.0861
H	0.7404	2.1933	0.0234
H	0.7717	−2.1607	0.0389
H	−1.7252	−2.1531	−0.0748
H	−2.9262	−0.0024	−0.1330
C	0.9468	0.0078	0.1906
H	0.8919	−0.0098	1.3354
O	2.2526	−0.0850	−0.2517
H	2.8011	0.6425	0.0665

o–H^+–C_6H_5OH

$E_{El} = -307.910\,528\,730\,E_h$
$E_{El+ZPE} = -307.794\,769\,E_h$

Atom	x	y	z
C	−1.8736	0.0737	0.0000
C	−1.0819	1.2561	0.0000
C	0.2993	1.2487	0.0000
C	0.9623	0.0263	0.0000
C	−1.2635	−1.1314	0.0000
H	−2.9501	0.1578	0.0000
H	−1.5880	2.2136	0.0000
H	0.8554	2.1768	0.0000
H	−1.8331	−2.0509	0.0000
O	2.2636	−0.1076	0.0000
H	2.7392	0.7380	0.0000
C	0.2100	−1.2475	0.0000
H	0.5406	−1.8520	0.8578
H	0.5406	−1.8520	−0.8578

i/o–H^+–C_6H_5OH TS

$E_{El} = -307.865\,093\,447\,E_h$
$E_{El+ZPE} = -307.752\,860\,E_h$

Atom	x	y	z
C	1.8521	0.0177	−0.0563
C	1.1446	1.2354	−0.0134
C	−0.2240	1.2484	0.0248
C	−0.9501	0.0016	0.0797
C	1.1949	−1.1993	−0.0241
H	2.9325	0.0383	−0.1016
H	1.6877	2.1697	−0.0341
H	−0.7786	2.1779	0.0297
H	1.7457	−2.1285	−0.0390
O	−2.2811	−0.0978	−0.1493
H	−2.7513	0.7275	0.0257
C	−0.1973	−1.2339	0.0088
H	−0.7624	−2.1562	0.0428
H	−0.6668	−0.3379	1.2109

o/m–H^+–C_6H_5OH TS

$E_{El} = -307.874\,568\,450\,E_h$
$E_{El+ZPE} = -307.762\,067\,E_h$

Atom	x	y	z
C	−1.8648	0.0842	−0.0277
C	0.9793	0.0125	−0.0103
C	0.2792	1.2149	0.0101
C	−1.1193	1.2440	−0.0024
H	−2.9437	0.1076	−0.0397
H	0.8272	2.1481	0.0427
H	−1.6189	2.2023	0.0103
C	0.2527	−1.1959	−0.0561
H	0.7692	−2.1453	−0.0893
O	2.3082	−0.1068	−0.0093
H	2.7625	0.7460	−0.0095
C	−1.1921	−1.1617	−0.0341
H	−1.7302	−2.0899	−0.1738
H	−0.6344	−1.2733	1.0598

$\boldsymbol{m}$–H^+–C_6H_5OH

$E_{\mathrm{El}} = -307.889\,102\,893\,E_{\mathrm{h}}$
$E_{\mathrm{El+ZPE}} = -307.775\,012\,E_{\mathrm{h}}$

Atom	x	y	z
C	−1.8512	0.1340	−0.0002
C	0.9962	−0.0268	0.0002
C	0.2937	1.1973	0.0002
C	−1.1063	1.2795	0.0000
H	−2.9321	0.1666	−0.0005
H	0.8632	2.1207	0.0004
H	−1.5770	2.2520	−0.0001
C	0.2682	−1.1952	0.0003
H	0.7814	−2.1478	0.0006
O	2.3335	−0.1115	0.0001
H	2.7711	0.7486	0.0009
C	−1.1905	−1.1676	0.0000
H	−1.5711	−1.7729	−0.8445
H	−1.5714	−1.7727	0.8445

$\boldsymbol{p}$–H^+–C_6H_5OH

$E_{\mathrm{El}} = -307.914\,941\,188\,E_{\mathrm{h}}$
$E_{\mathrm{El+ZPE}} = -307.799\,010\,E_{\mathrm{h}}$

Atom	x	y	z
O	−2.2809	0.1064	0.0000
H	−2.7298	−0.7536	0.0000
C	−0.9776	0.0185	0.0002
C	−0.3038	−1.2374	0.0001
C	−0.2698	1.2538	0.0000
C	1.0464	−1.2597	0.0001
H	−0.8833	−2.1518	0.0001
C	1.0800	1.2372	0.0000
H	−0.8418	2.1705	0.0001
H	1.5741	−2.2046	0.0001
H	1.6346	2.1664	0.0000
C	1.8456	−0.0222	−0.0001
H	2.5417	−0.0339	−0.8542
H	2.5423	−0.0339	0.8535

$\boldsymbol{m/p}$–H^+–C_6H_5OH TS

$E_{\mathrm{El}} = -307.876\,594\,617\,E_{\mathrm{h}}$
$E_{\mathrm{El+ZPE}} = -307.764\,032\,E_{\mathrm{h}}$

Atom	x	y	z
C	−1.8406	0.0813	−0.0559
C	0.9807	−0.0289	−0.0049
C	0.2859	1.2071	−0.0004
C	−1.0878	1.2648	−0.0143
H	−2.9205	0.0875	−0.0868
H	0.8549	2.1286	0.0211
H	−1.5945	2.2185	−0.0106
C	0.2683	−1.2175	−0.0278
H	0.7930	−2.1612	−0.0453
O	2.3103	−0.1085	0.0071
H	2.7434	0.7547	0.0075
C	−1.1461	−1.1844	−0.0330
H	−1.7098	−2.0962	−0.1820
H	−1.5262	−0.7592	1.0594

$\boldsymbol{i/O}$–H^+–C_6H_5OH TS

$E_{\mathrm{El}} = -307.819\,156\,770\,E_{\mathrm{h}}$
$E_{\mathrm{El+ZPE}} = -307.708\,656\,E_{\mathrm{h}}$

Atom	x	y	z
C	1.2016	−1.2109	−0.0012
C	−0.1801	−1.2384	−0.0468
C	−0.8509	−0.0044	−0.0039
C	−0.1944	1.2393	−0.0361
C	1.1860	1.2253	−0.0011
C	1.8764	0.0100	0.0141
H	1.7537	−2.1395	0.0005
H	−0.7419	−2.1612	−0.0864
H	−0.7489	2.1684	−0.0545
H	1.7285	2.1595	0.0053
H	2.9574	0.0177	0.0313
O	−2.3488	−0.1025	−0.0345
H	−2.7343	0.7820	−0.1658
H	−1.5765	−0.0679	1.0243

o/O–H^+–C_6H_5OH TS

$E_{El} = -307.815\,581\,444\,E_h$
$E_{El+ZPE} = -307.706\,336\,E_h$

Atom	x	y	z
C	−1.8563	−0.1076	0.0933
C	−1.0940	−1.2587	0.0362
C	0.8566	0.1420	−0.1632
C	0.1326	1.3013	−0.0953
C	−1.2532	1.1524	0.0035
H	−2.9300	−0.1759	0.1900
H	−1.5582	−2.2343	0.0382
H	0.6044	2.2737	−0.0737
H	−1.8689	2.0402	0.0509
C	0.3042	−1.1521	−0.1184
H	1.5285	−0.9708	0.6578
H	0.8747	−2.0039	−0.4950
O	2.2681	−0.0172	0.0526
H	2.6443	0.6685	0.6281

$C_6H_5OH_2^+$

$E_{El} = -307.885\,229\,467\,E_h$
$E_{El+ZPE} = -307.769\,277\,E_h$

Atom	x	y	z
C	1.1933	1.2260	0.0000
C	−0.1994	1.2224	0.0000
C	−0.7976	−0.0159	0.0000
C	−0.1634	−1.2337	0.0000
C	1.2293	−1.1949	0.0000
C	1.8971	0.0254	0.0000
H	1.7191	2.1697	0.0000
H	−0.7651	2.1439	0.0000
H	−0.7124	−2.1637	0.0000
H	1.7832	−2.1224	0.0000
H	2.9775	0.0421	0.0000
O	−2.3043	−0.0802	0.0000
H	−2.7209	0.2845	−0.8022
H	−2.7209	0.2845	0.8021

C_6H_5OH

$E_{El} = -307.590\,311\,156\,E_h$
$E_{El+ZPE} = -307.485\,835\,E_h$

Atom	x	y	z
C	1.8528	0.0274	0.0000
C	1.1310	1.2146	0.0000
C	−0.2592	1.1951	0.0000
C	−0.9340	−0.0231	0.0000
C	−0.2182	−1.2177	0.0000
C	1.1691	−1.1852	0.0000
H	2.9334	0.0455	0.0000
H	1.6478	2.1648	0.0000
H	−0.8185	2.1234	0.0000
H	−0.7591	−2.1535	0.0000
H	1.7199	−2.1162	0.0000
O	−2.2994	−0.1069	0.0000
H	−2.6802	0.7768	0.0000

References

(S1) Hunter, E. P. L.; Lias, S. G. Evaluated Gas Phase Basicities and Proton Affinities of Molecules: An Update. *J. Phys. Chem. Ref. Data* **1998**, *27*, 413–656.

(S2) Reutt, J. E.; Wang, L. S.; Lee, Y. T.; Shirley, D. A. Molecular beam photoelectron spectroscopy and femtosecond intramolecular dynamics of H_2O^+ and /chD2O+. *J. Chem. Phys.* **1986**, *85*, 6928–6939.

(S3) Ashmore, F. S.; Burgess, A. R. Study of some medium size alcohols and hydroperoxides by photoelectron spectroscopy. *J. Chem. Soc., Faraday Trans. 2* **1977**, *73*, 1247–1261.

(S4) Brown, R. S. A Photoelectron Investigation of the Peroxide Bond. *Can. J. Chem.* **1975**, *53*, 3439–3447.

(S5) Nemeth, G.; Selzle, H.; Schlag, E. Magnetic ZEKE experiments with mass analysis. *Chem. Phys. Lett.* **1993**, *215*, 151–155.
